# Decoding IFN-mediated immunity and cell dynamics in viral encephalitis: Insights from coxsackievirus B3 infection

**DOI:** 10.1016/j.isci.2025.114324

**Published:** 2025-12-03

**Authors:** Lisa Marie Stach, Lisa Gerarda Maria Huis in ‘t Veld, Theres Schaub, Marina Jendrach, Marta Ornaghi, Marius Schwabenland, Antje Beling, Sandra Pinkert

**Affiliations:** 1 Charité – Universitätsmedizin Berlin, Corporate Member of Freie Universität Berlin and Humboldt-Universität zu Berlin, Institute of Biochemistry, 10117 Berlin, Germany; 2 Charité – Universitätsmedizin Berlin, corporate Member of Freie Universität Berlin and Humboldt-Universität zu Berlin, Institute of Cell Biology and Neurobiology, 10117 Berlin, Germany; 3 Charité – Universitätsmedizin Berlin, corporate Member of Freie Universität Berlin and Humboldt-Universität zu Berlin, Department of Neuropathology, 10117 Berlin, Germany; 4 Department of Neuropathology, Medical Centre-University of Freiburg, 79106 Freiburg, Germany; 5 Deutsches Zentrum für Herz-Kreislauf-Forschung, Partner Site Berlin, 10117 Berlin, Germany

**Keywords:** Neuroscience, Immunology, Virology

## Abstract

This study presents an *in vivo* model of coxsackievirus B3 infection in the brain to examine interferon (IFN) responses and effects on tissue-resident immune cells. Primary cell culture studies revealed that neurons exhibited substantial viral replication with a limited IFN response, whereas microglia, which showed no viral replication, displayed strong immune activation. *In vivo* analysis captured IFN responses and immune cell dynamics across both acute and chronic inflammation phases. During the acute stage, IFN responses intensified in a dose-dependent manner, with upregulated IFN-stimulated genes (ISGs), increased ISGylation, and microglial activation. Chemokine release coincided with the infiltration of monocytes and T cells in the injured brain. In the chronic phase, viral RNA was undetectable, yet flow cytometry showed persistent T cell presence and low-level microglial activation, indicating ongoing inflammation. This model provides a valuable platform for investigating IFN responses and immune cell interactions in central nervous system (CNS) viral infections and neuroinflammatory conditions.

## Highlights

• Microglia show a robust antiviral immune response when treated with coxsackievirus
• In contrast, neurons infected with coxsackievirus exhibit a weaker immune response
• Acute coxsackievirus infection of mice leads to a potent immune response in the CNS
• Chronic post-viral phase shows low-grade T cell abundance in the CNS

## Graphical abstract

**Figure:**
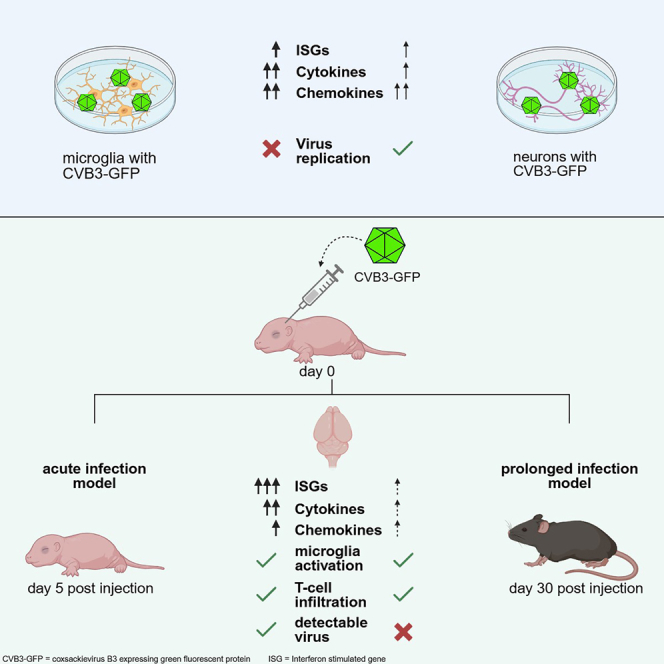

## Introduction

Viral infections of the central nervous system (CNS) encompass a broad spectrum of acute and chronic pathologies, ranging from mild to life-threatening conditions. Among these, enteroviruses are particularly notable for their ability to invade the CNS, leading to serious conditions such as aseptic meningitis and encephalitis, which manifest in neurological symptoms such as ataxia and paralysis. These infections pose a substantial risk of morbidity and mortality, especially in neonates, who are particularly vulnerable to viral assaults on the CNS.^1^^,^^2^^,^^3^^,^^4^^,^^5^^,^^6^^,^^7^ Moreover, *in utero* exposure to enteroviruses can lead to spontaneous abortion or severe neurodevelopmental impairments, further highlighting the devastating impact of these infections.^2^^,^^8^^,^^9^ Among enteroviruses, coxsackieviruses are a frequent cause of enteroviral CNS infections in newborns, with the neonatal nervous system being particularly susceptible to their effects.^10^^,^^11^^,^^12^ Coxsackieviruses are categorized into two subgroups, A and B, based on their pathogenic profiles in neonatal mice.^13^ Group B coxsackieviruses (CVB), including CVB3, exhibit marked neurotropism, which has been demonstrated in both murine models^10^^,^^11^^,^^12^ and in human infections. In humans, CVB infections have been associated with neurological conditions, such as meningoencephalitis and upper motor neuron injury.^10^^,^^11^^,^^14^ While most coxsackievirus infections are acute, they have also been implicated in long-term neurological conditions, including adult-onset schizophrenia, encephalitis lethargica, and amyotrophic lateral sclerosis.^8^^,^^15^^,^^16^ Previous studies utilizing CVB3 as a model pathogen for the intracerebroventricular infection of the CNS in mice demonstrated that the virus exhibits a distinct tropism for neurons, with the initial infection of neuronal progenitor cells in the subventricular zone (SVZ).^10^^,^^11^ Infected progenitor cells continue migrating along their designated pathways, ultimately reaching regions such as the cerebral cortex and olfactory bulb.^10^^,^^11^ As the infection progresses, viral proteins localize primarily to mature neurons, while astrocytes remain largely unaffected. The susceptibility to CNS infection by CVB3 is age-dependent, with neonatal mice being vulnerable, whereas susceptibility declines with age,^10^ reminiscent of the age-dependent pattern seen in humans. Although the infectious virus is no longer detectable by 30 days post-infection, CVB3 RNA can persist in the brain, consistent with reports of prolonged CVB RNA presence in both murine and human tissues.^17^^,^^18^^,^^19^^,^^20^

Microglia, the resident macrophages of the CNS, play a pivotal role in controlling viral infections and are therefore likely to contribute significantly to the immune response against CVB3. Originating from embryonic yolk sac precursors, microglia constitute up to 10% of CNS cells in adults.^21^ As key components of the innate immune response, microglia continuously monitor the CNS for pathogens or damage and are particularly adept at detecting viral infections.^22^^,^^23^ Notably, microglia can respond to CNS insults within minutes, rapidly deploying defense mechanisms to combat infections.^24^ Infection of neonatal mice with CVB3 has been shown to induce microglial activation, as indicated by morphological changes and phagocytosis activity.^20^ Studies using microglial depletion models in other infection models have consistently shown that the absence of microglia leads to increased viral loads in the CNS, underscoring their essential antiviral role.^25^^,^^26^^,^^27^^,^^28^^,^^29^ While the specific mechanisms of CVB3 recognition in the CNS remain unclear, it is known that CVB3 infection in the heart is detected by the immune system via Toll-like receptors (TLRs) 7 and TLR3.^30^^,^^31^ Moreover, microglia are known to detect viral RNA through these pattern recognition receptors (PRRs) and initiate type I interferon (IFN) signaling cascades.^32^^,^^33^ As principal producers of type I IFNs within the CNS during viral infections, microglia play a pivotal role in orchestrating the antiviral response; however, their specific function in CVB3 infection remains unclear.^34^ The release of type I IFNs triggers the upregulation of hundreds of interferon-stimulated genes (ISGs) that collectively mount a robust antiviral response.^35^^,^^36^^,^^37^ Notably, microglial-derived IFNs also stimulate antiviral responses in other CNS-resident cells, including neurons and astrocytes, thereby reinforcing the brain’s overall immune defense.^38^^,^^39^ However, prolonged or excessive activation of microglia can lead to neuroinflammation and contribute to neurodegenerative processes, a phenomenon referred to as “microgliopathy”.^40^ Despite these risks, constitutive IFN signaling appears essential for maintaining microglia in a primed state, allowing them to respond swiftly to infections without tipping into pathological overactivation.^41^ In addition to their direct antiviral roles, microglia secrete pro-inflammatory cytokines, such as TNF-α and IL-1β, further amplifying the immune response within the CNS. Microglia are rapidly recruited to affected areas, such as infected neurons, where they engage in additional antiviral functions, such as phagocytosis and autophagy.^25^ Moreover, microglia contribute to neuronal repair and the re-establishment of CNS homeostasis following infection, highlighting their multifaceted role in maintaining CNS integrity.^42^

In this study, we employ an *in vivo* model of CVB3 infection, with a specific focus on characterizing the IFN response in neurons, the primary target of infection, and assessing microglial activation. By integrating *in vitro* data from primary neuronal and microglial cultures with *in vivo* findings on viral replication, IFN-driven responses, and immune cell infiltration, we provide a comprehensive assessment of how tissue-resident immune cells contribute to both the acute phase of CVB3 infection and chronic inflammation. Our study reveals that during the acute phase, robust IFN responses are marked by strong ISG activation and chemokine-driven recruitment of immune cells. However, we also observe chronic inflammation, characterized by T cell infiltration and sustained microglial activation, despite the absence of detectable viral genomes.

## Results

### Neuronal response to CVB3 and Toll-like receptor agonists: modest cytokine and interferon-induced gene activation

**Figure 1:**
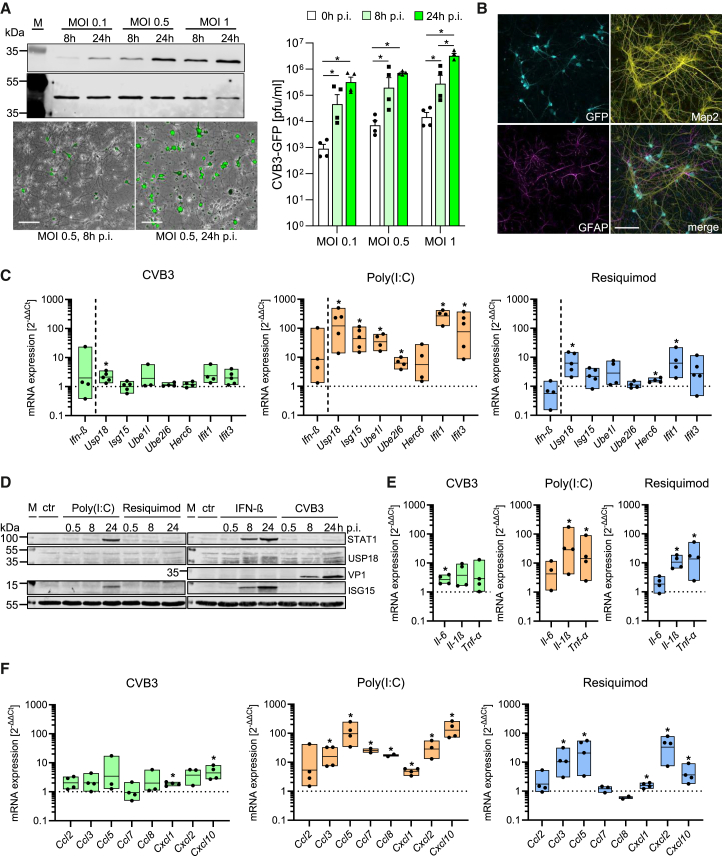
CVB3 infection in primary neuronal cells triggers viral replication and modest activation of cytokines, chemokines, and ISGs (A) Primary neuronal cultures derived from embryonic day 16 (E16) mice were infected with GFP-expressing CVB3 at MOI of 0.1, 0.5, or 1, and analyzed at 8- and 24-h post-infection (h p.i.). Upper panel: Viral replication was assessed by the quantification of the viral capsid protein VP1 using Western blotting with an anti-VP1 antibody. Actin served as the loading control. Lower panel: GFP expression in infected cells was visualized by fluorescence microscopy at 8 and 24 h p.i.; the corresponding scale bar (100 μm) is indicated. Additionally, infectious virus titers were quantified at 0, 8, and 24 h p.i. by plaque assay (*n* = 2 experiments with 2 biological replicates; data were log-transformed, plotted as individual points, and results were presented as mean ± SEM. Data were analyzed by two-way ANOVA, with significant results (*p* < 0.05) indicated by an asterisk (∗). (B) Confocal microscopy of infected neuronal cultures, stained with antibodies Map2 (neuronal marker) and GFAP (astrocyte marker), illustrating GFP expression predominantly in Map2^+^ neurons. Corresponding scale bar (50 μm) is indicated. (C) Quantitative RT-PCR analysis of *Ifn-β* and ISG expression 24 h post-infection with CVB3 (MOI 0.5) or after stimulation with Poly(I:C) (50 μg/mL) and Resiquimod (10 μg/mL). Data were normalized to untreated controls and log-transformed for statistical analysis. Data were plotted as individual points, and results were presented as mean ± SEM. One-sample *t*-tests were performed (CVB3: *n* ≥ 3, Poly(I:C): *n* ≥ 4, Resiquimod: *n* ≥ 4), with significant results (*p* < 0.05) indicated by an asterisk (∗). (D) Immunoblot analysis of ISG expression in neuronal cultures treated with CVB3 (MOI 0.5), Poly(I:C) (50 μg/mL), Resiquimod (10 μg/mL), or IFN-β (100 U/mL). Western blots were probed with the indicated antibodies, with tubulin used as a loading control. A representative blot from three independent experiments is shown. (E) Quantitative RT-PCR analysis of cytokine expression 24 h post-infection with CVB3 (MOI 0.5) or following stimulation with Poly(I:C) (50 μg/mL) and Resiquimod (10 μg/mL). Results were normalized to untreated controls, log-transformed, and analyzed using one-sample *t*-tests (CVB3: *n* = 4, Poly(I:C): *n* ≥ 3, Resiquimod: *n* = 4). Data were plotted as individual points, and results were presented as mean ± SEM. Significant results (*p* < 0.05) are denoted by an asterisk (∗). (F) Chemokine expression was measured by quantitative RT-PCR 24 h post-infection with CVB3 (MOI 0.5) or after stimulation with Poly(I:C) (50 μg/mL) and Resiquimod (10 μg/mL). Data were normalized to untreated controls and log-transformed for statistical analysis. Data were plotted as individual points, and results were presented as mean ± SEM. One-sample *t*-tests were performed (CVB3: *n* ≥ 3, Poly(I:C): *n* ≥ 2, Resiquimod: *n* ≥ 2), with significant findings (*p* < 0.05) marked by an asterisk (∗).

To investigate how CVB3 infection affects neuronal homeostasis, we first examined viral replication in primary neuronal cell cultures. Western blot detection of VP1, a viral capsid protein, confirmed viral replication, with levels increasing over time, particularly at higher multiplicities of infection (MOI 0.5 and 1). Furthermore, active viral replication in neuronal culture was evidenced by an increase in GFP expression and an increase in infectious virus following infection with the recombinant GFP-expressing CVB3 (Figure 1A). Immunofluorescence staining corroborated these findings, showing GFP expression as evidence for *de novo* protein expression in infected cells predominantly in neurons, as marked by MAP2, while astrocytes, marked by GFAP, exhibited no GFP signals (Figure 1B). These results demonstrate that CVB3 replication occurs mainly in neurons. Thereby, the additional expression of GFP resulted only in a slightly attenuated replication efficacy in neuronal cell culture compared to wild-type CVB3 (Figure S1A). Next, we assessed the immune activation profile of neuronal cells during CVB3 infection in comparison to their response to two TLR agonists, mimicking viral RNA recognition by TLR3 (Poly(I:C)) and TLR7 (Resiquimod). Thus, the 24 h time point was selected based on mRNA expression kinetics, viral titer, and cell viability (Figures S1A, S1B, and 1A). Despite significant viral replication in neurons, CVB3 induced a weak type I interferon (IFN) response, as indicated by negligible expression levels of *Ifn-β* as well as low expression levels of the IFN-induced genes (ISGs), such as *IFN-stimulated gene 15 (Isg15), ubiquitin specific peptidase 18 (Usp18)*, and *IFN-induced protein with tetratricopeptide repeats (Ifit)* (Figure 1C). In contrast, Poly(I:C) treatment led to the substantial upregulation of ISGs, such as *Isg15, Ifit1,* and *Ifit3* (Figure 1C), whereas Resiquimod failed to elicit a comparable response in neuronal cells. These data suggest that TLR3-mediated signaling via Poly(I:C) is effective in promoting IFN responses in neuronal cells, with CVB3 infection showing more subtle effects. Western blot analysis confirmed that CVB3 infection in neuronal cells does not elicit a strong IFN response despite increased VP1 expression, while Poly(I:C) and IFN-β lead to a strong induction of STAT1 and ISG15 (Figure 1D). Cytokine and chemokine mRNA levels were also analyzed. CVB3 infection resulted in a modest increase in chemokines, particularly *Cxcl10*, but this response was markedly weaker than the robust upregulation observed after Poly(I:C) stimulation (5-fold vs. 150-fold, Figures 1E and 1F). Thus, while Poly(I:C) treatment induced a strong immune response, CVB3 elicited a relatively limited immune activation in neuronal cells.

### Microglial activation by CVB3 and Toll-like receptor agonists: strong interferon-I and cytokine response

**Figure 2:**
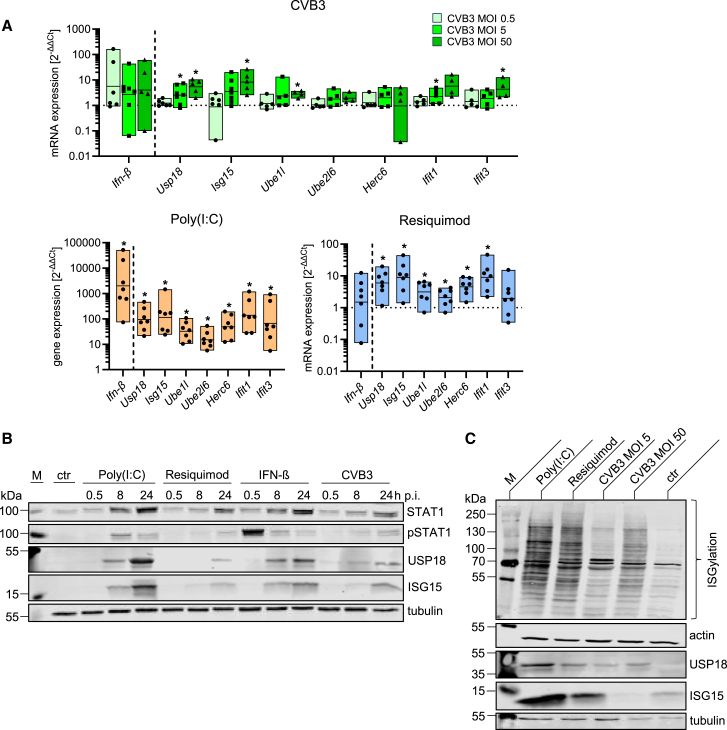
Stimulation of microglia with TLR agonists and CVB3 increases ISG mRNA expression and ISGylation (A) mRNA expression of *Ifn-β* and ISGs in primary microglia 24 h after stimulation with CVB3 (MOI 0.5, 5, or 50), Poly(I:C) (50 μg/mL), or Resiquimod (10 μg/mL). Data were normalized to untreated controls and logarithmically transformed. Data were plotted as individual points and results were presented as mean ± SEM. Statistical significance was assessed using one-sample *t*-tests (CVB3: *n* ≥ 4, Poly(I:C): *n* = 7, Resiquimod: *n* = 7), with significant results (*p* < 0.05) marked by an asterisk (∗). (B) Microglia were treated with CVB3 (MOI 50), Poly(I:C) (50 μg/mL), Resiquimod (10 μg/mL), or IFN-β (100 U/mL) and harvested at the indicated time points. Expression of ISGs (STAT1, p-STAT1, ISG15, USP18) was assessed by immunoblotting. Tubulin served as a loading control. A representative blot from three independent experiments is shown. (C) Microglia were treated for 24 h with CVB3 (MOI 50), Poly(I:C) (50 μg/mL), Resiquimod (10 μg/mL), or IFN-β (100 U/mL). ISG15, USP18, and ISGylation were analyzed via immunoblotting. Tubulin and actin served as loading controls. A representative blot from three independent experiments is shown.

We next assessed the immune response of microglia to CVB3 infection in comparison to the TLR agonists Poly(I:C) and Resiquimod using primary microglia cultured from neonatal or adult mice (Figures S2A and S2B). Notably, even at high MOIs, incubation with CVB3 did not result in detectable viral replication, as evidenced by the absence of viral protein expression and even a decrease in infectious viral particles (Figures S2C and S2D). Consistently, in comparison to neuronal cells, microglia had much lower expression levels of the CVB3 receptor, *coxsackievirus and adenovirus receptor (Car)* (Figures S2E). In neonatal primary microglia, incubation with CVB3 at a high MOI led to a significant upregulation of ISGs, including *Usp18, Isg15*, and *Ifits* after 24 h, similar to the response triggered by the TLR7 agonist Resiquimod (Figure 2A). TLR3 agonist Poly(I:C) induced a more than 10-fold higher expression of these ISGs with the administered doses. At the protein level, there were time-dependent increases in USP18, ISG15, and STAT1 across all stimulations, although the responses were less pronounced for CVB3 and Resiquimod (Figure 2B). STAT1 phosphorylation, indicative of ongoing IFNAR signaling, was observed consistently under all conditions, and occurred quickly upon direct stimulation by IFN-β. Protein ISGylation, evidenced by the increased detection of high-molecular-weight protein bands using an ISG15 antibody, as well as the activation of the ISGylation machinery (Ube1L, Ube2L6, and Herc6), was also observed in CVB3-treated microglia (Figure 2C). Similar to ISG expression, Poly(I:C) and Resiquimod induced stronger ISGylation than CVB3 at these doses. Compared to other neuronal cells, microglia demonstrate a markedly stronger response to various stimuli, with Poly(I:C) inducing the most robust ISGylation, followed by Resiquimod. Notably, even CVB3 incubation is competent to induce an ISG response in microglia, underscoring their heightened sensitivity to both synthetic immune activators and viral infection.

**Figure 3:**
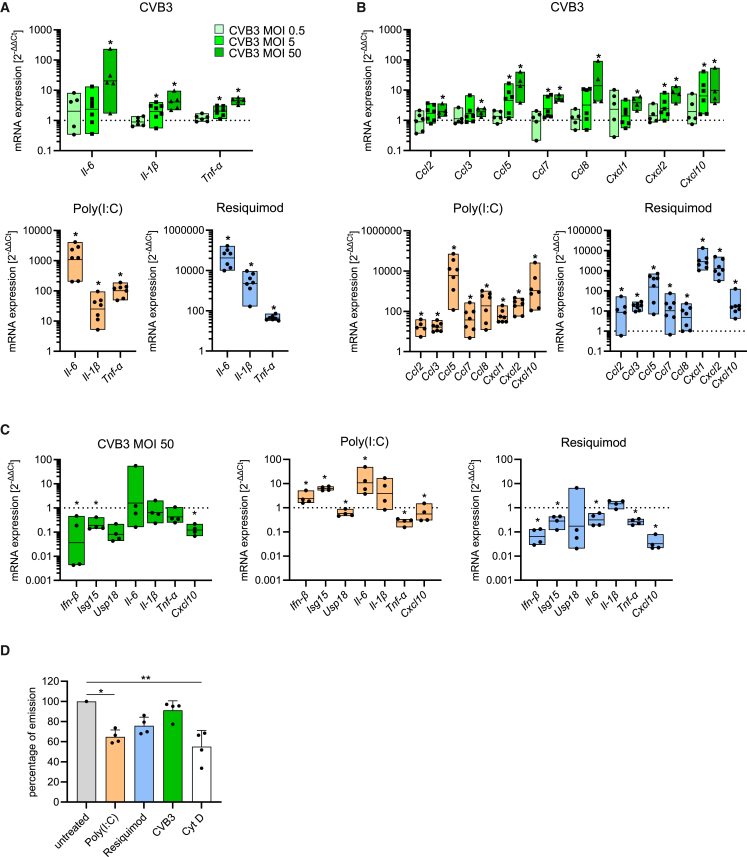
TLR agonists and CVB3 stimulate microglial activation with increased cytokine and chemokine expression (A) Cytokine expression 24 h after primary microglia were stimulated with CVB3 (MOI 0.5, 5, or 50) - upper graph -, Poly(I:C) (50 μg/mL), or Resiquimod (10 μg/mL) - lower graph -, as analyzed by quantitative RT-PCR. Data were normalized to untreated controls and logarithmically transformed. Statistical significance was assessed using one-sample *t*-tests (CVB3: *n* ≥ 5, Poly(I:C): *n* = 7, Resiquimod: *n* = 7). Significant results (*p* < 0.05) are marked with an asterisk (∗). (B) Chemokine expression 24 h after primary microglia stimulation with CVB3 (MOI 0.5, 5, or 50) - upper graph -, Poly(I:C) (50 μg/mL), or Resiquimod (10 μg/mL) - lower graphs - analyzed by quantitative RT-PCR. Data were normalized to untreated controls and logarithmically transformed. Statistical analysis was performed using one-sample *t*-tests (CVB3: *n* ≥ 4, Poly(I:C): *n* ≥ 5, Resiquimod: *n* ≥ 5). Significant results (*p* < 0.05) are marked with an asterisk (∗). (C) Comparison of mRNA expression of different primary microglia cultures. Neonatal microglia (*n* = 5) and adult microglia (*n* = 4) were stimulated with CVB3 (MOI 50), Poly(I:C) (50 μg/mL), or Resiquimod (10 μg/mL), and mRNA expression was analyzed by quantitative RT-PCR 24 h post stimulation. The mRNA expression level of adult microglia is shown relative to neonatal microglia (set as 1). Statistical analysis was conducted using one-sample *t* test. Significant results (*p* < 0.05) are marked with an asterisk (∗). (D) Primary microglia were stimulated with Poly(I:C) (50 μg/mL), Resiquimod (10 μg/mL), or CVB3 (MOI 50) for 24 h. Phagocytosis was then assessed using a phagocytosis assay. Cytochalasin D was used as a negative control. Data were normalized to untreated controls, and statistical analysis was performed using ordinary one-way ANOVA (*n* = 4). Significant results (*p* < 0.05) are marked with an asterisk (∗). All data were plotted as individual points, and results were presented as mean ± SEM.

While the IFN response is the hallmark reaction following viral recognition, leading to the production of direct antiviral interferon-stimulated genes (ISGs), pro-inflammatory cytokines and chemokines play an equally critical role in orchestrating a comprehensive immune response. These molecules promote cellular communication, recruit immune cells to the site of infection, and facilitate tissue repair. To further investigate whether CVB3 could stimulate cytokine and chemokine production in microglia, we employed Poly(I:C) and Resiquimod as controls, both of which are known to robustly induce these responses. CVB3 incubation at a high MOI led to increased mRNA expression of key cytokines and chemokines, notably *Il-6, Ccl5, Ccl8,* and *Cxcl10* (Figures 3A and 3B). However, this increase was relatively modest compared to the potent induction observed with Poly(I:C) and Resiquimod, which significantly upregulated mRNA levels of *Il-6, Il-1β*, and *Tnf-α*. In contrast to ISG expression, where Poly(I:C) elicited the strongest response, Resiquimod triggered the highest upregulation of *Il-6* (CVB3: 60-fold vs. Poly(I:C): 1,750-fold vs. Resiquimod: 65,000-fold) and *Il-1β* (CVB3: 5-fold vs. Poly(I:C): 36-fold vs. Resiquimod: 3,780-fold). The analysis of chemokine expression revealed marked variability depending on the stimulus, with Poly(I:C) producing the highest expression of *Ccl5* (20,000-fold) and *Cxcl10* (4,800-fold), while Resiquimod strongly induced *Cxcl1* (4,000-fold) and *Cxcl2* (1,900-fold). By comparison, CVB3 treatment at MOI 50 resulted in a more modest 10-fold increase in mRNA levels of *Ccl5, Ccl8*, and *Cxcl10* (Figure 3B). We also generated mRNA expression profiles for selected targets in primary microglia that we isolated from adult brain tissue and compared these profiles with the ones obtained from cells of neonatal origin. As shown in Figure 3C, we found only minor differences in the mRNA expression profiles for *Ifn-β*, *Isg15*, *Usp18*, *Il-6*, *Il-1β*, *TNF-α,* and *Cxcl10,* upon CVB3, Poly(I:C), or Resiquimod treatment. However, it should be noted that a direct comparison between viral infection and pure TLR stimulation is limited, as they represent different modes of immune activation. In addition to evaluating cytokine and chemokine production, we examined the functional consequences of microglial activation, focusing on cells from neonatal origin. The pHrodo assay, used to assess phagocytic activity, revealed that Poly(I:C) stimulation led to a reduction in phagocytosis (Figure 3D). In contrast, CVB3 and Resiquimod treatments had less impact on phagocytic activity. In summary, CVB3 infection serves as a potent stimulus for microglia activation, evidenced by cytokine and chemokine expression. As expected, stimulation with the TLR ligands Poly(I:C) and Resiquimod elicited even stronger inflammatory responses. This supports the argument that CVB3 may directly contribute to microglia activation, and this process potentially involves one of these TLR pathways.

### Intracerebroventricular CVB3 infection triggers immune activation and immune cell infiltration

Building on our *in vitro* analysis of primary neuronal cells and microglia, which demonstrated robust viral replication in neurons alongside a relatively muted IFN response, coupled with strong microglial activation upon TLR stimulation and high-dose CVB3 infection, we extended our investigation to characterize the IFN response and inflammatory dynamics *in vivo* during CVB3 infection. Special focus was placed on microglial activation and the infiltration of peripheral immune cells into the CNS.

**Figure 4:**
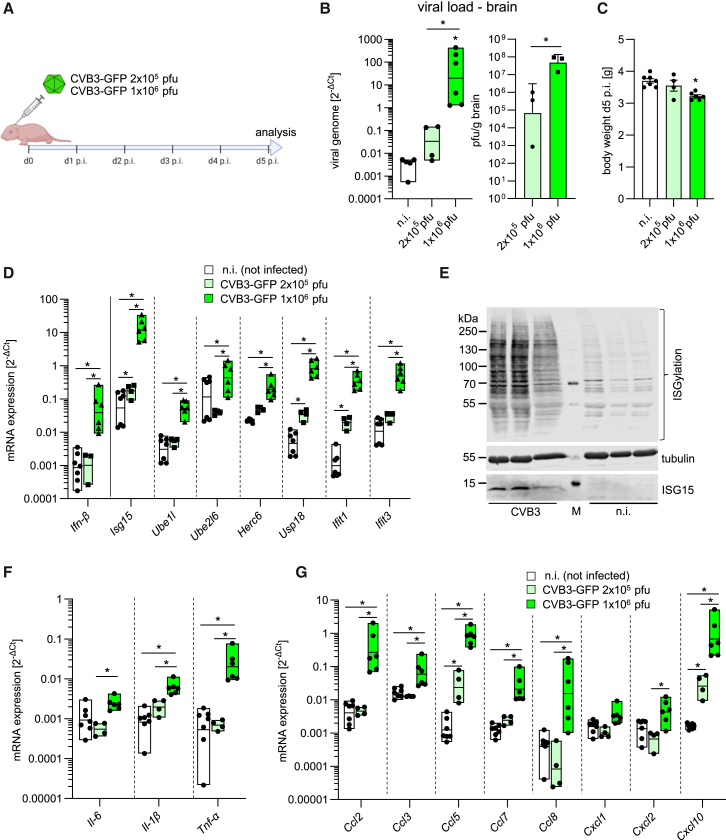
Intracerebroventricular CVB3 infection of neonatal mice triggers immune activation with elevated ISG, cytokine, and chemokine expression (A) Newborn mice (P0) were intracerebroventricularly injected with either 2 × 10^5^ pfu (*n* = 4) or 1 × 10^6^ pfu (*n* = 6) of CVB3 expressing GFP. Mice were sacrificed and analyzed 5 days post-infection. (B) CVB3 genome levels (left, data log-transformed and analyzed by one-way ANOVA) and infectious virus titers (right, log-transformed data analyzed by *t* test) were quantified in the brain by RT-PCR and plaque assay, respectively. (C) Body weight was measured 5 days post-infection (n.i., *n* = 7; one-way ANOVA). (D) mRNA expression of various ISGs in infected and uninfected mice, quantified by RT-PCR. Data were log-transformed and analyzed by two-way ANOVA. Significant results (*p* < 0.05) are marked with an asterisk (∗). (E) Western blot analysis of ISG15 and ISGylation in brain lysates from infected (1 × 10^6^ pfu) and uninfected animals. Tubulin was used as a loading control. (F and G) mRNA expression of pro-inflammatory cytokines (F) and chemokines (G) in brain tissue from infected and uninfected mice, quantified by RT-PCR. Data were logarithmically transformed and analyzed by two-way ANOVA. Significant results (*p* < 0.05) are marked with an asterisk (∗). All data were plotted as individual points, and results were presented as mean ± SEM.

First, we infected C57BL/6J neonates with CVB3-GFP and evaluated both the IFN and inflammatory responses in the brain on day 5 post-infection (Figure 4A), a time point during the activated inflammatory phase in this encephalitis model.^20^ Viral genome was quantified through RT-qPCR (Figure 4B, left panel), and by plaque assays that quantified infectious particles in brain tissue (Figure 4B, right panel). As expected, mice infected with a high viral dose (1 × 10^6^ pfu) exhibited significantly greater viral loads compared to those infected with a lower dose (2 × 10^5^ pfu). After 5 days, a slight reduction in body weight was detected in mice receiving the higher dose (Figure 4C), without further signs of functional deterioration in any of the infected pups during this phase. The acute stage was marked by a strong IFN response, evidenced by elevated mRNA and protein levels of antiviral effectors. The RT-qPCR analysis of mice infected with the higher viral dose revealed substantial upregulation of *Isg15* and its conjugation machinery components, including *Ube1l, Ube2l6*, and *Herc6* (Figure 4D). Concurrently, IFN-stimulated genes (ISGs), such as *Ifit1* and *Ifit3*, were significantly elevated, indicating a robust antiviral response. Protein analysis corroborated these findings, demonstrating heightened ISG15 levels, and consistent with the upregulation of the ISG15 conjugation machinery, a marked increase in ISGylation was observed compared to non-infected controls, further confirming a potent IFN-driven antiviral response (Figure 4E). Regarding the proinflammatory response, *Tnf-α* exhibited the most pronounced upregulation, suggesting its central role in driving the inflammatory response to CVB3 infection (Figure 4F). Additionally, there was moderate induction of *Il-1β* and *Il-6*, though their levels were less prominently elevated. Chemokine production was notably elevated, with *Ccl2 (Mcp-1)*, *Ccl5 (Rantes)*, and *Cxcl10 (Ip-10)* significantly upregulated, which are crucial for recruiting monocytes and T cells to the CNS (Figure 4G).

**Figure 5:**
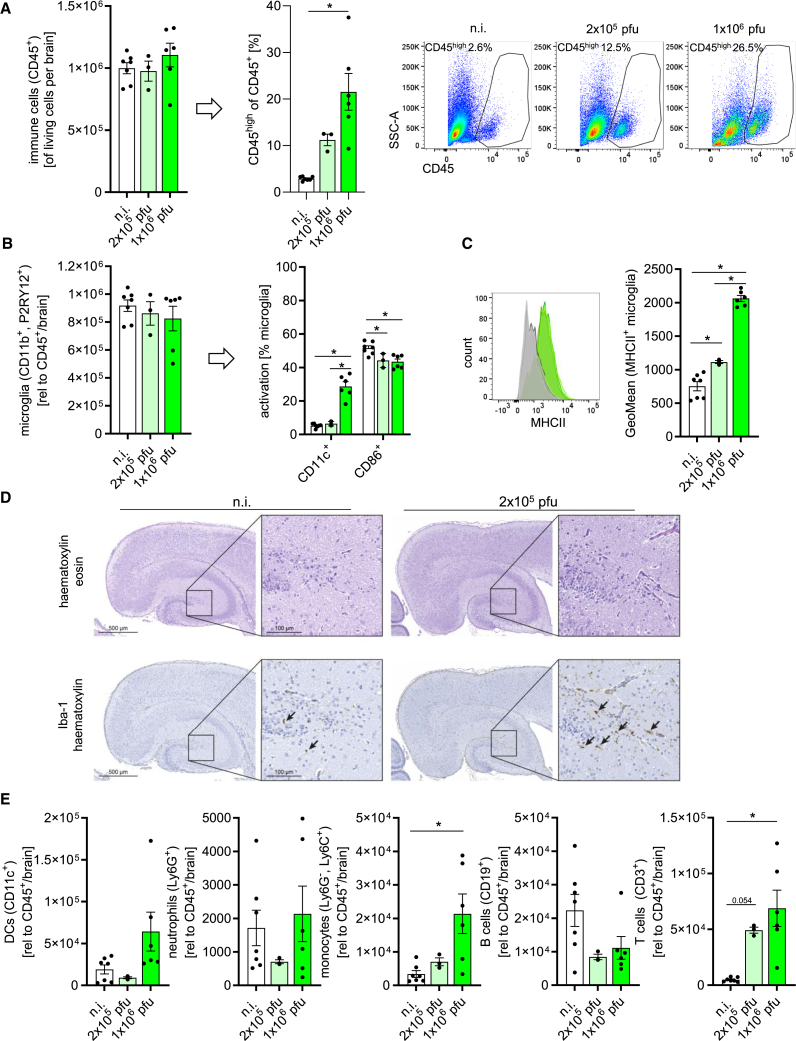
Enhanced T cell presence and microglial activation in CVB3-infected neonatal mouse brains Newborn mice (P0) were intracerebroventricularly injected with 2 × 10^5^ pfu (*n* = 3) or 1 × 10^6^ pfu (*n* = 6) of CVB3 expressing GFP, with uninfected controls (n.i., *n* = 7). Mice were sacrificed on day 5 post-infection, and immune cells were isolated from brain tissue for flow cytometry analysis. (A) Quantification of CD45^+^ immune cells and the percentage of CD45^high^ cells within this population by flow cytometry (one-way ANOVA). Histograms on the right represent one mouse from each group. (B) Left – Quantification of tissue-resident microglia (CD45^+^CD11b^+^P2RY12^+^), with further analysis of activation markers shown on the right. Statistical analysis was performed using one-way ANOVA. Significant results (*p* < 0.05) are marked with an asterisk (∗). (C) Left – Histogram overlay of MHC class II^+^ microglia from infected (1 × 10^6^ pfu, *n* = 3) and uninfected mice (*n* = 4). Right – geometric Mean fluorescence intensity (GeoMean) of MHC class II on microglia in infected and uninfected mice at day 5 post-infection. Statistical analysis was performed using one-way ANOVA. Significant results (*p* < 0.05) are marked with an asterisk (∗). (D) Histology of brain sections of infected (2 × 10^5^ pfu) and uninfected mice stained with hematoxylin and eosin or hematoxylin and Iba-1. Scale bars represent 500 μm for overview and 100 μm for zoom. Arrows indicate the Iba-1 positive microglia. Sections shown originate from one representative animal for each group. (E) Quantification of tissue-resident and infiltrating immune cells by flow cytometry at day 5 post-infection. Statistical analysis was performed using one-way ANOVA. Significant results (*p* < 0.05) are marked with an asterisk (∗). All data were plotted as individual points, and results were presented as mean ± SEM.

Flow cytometry provided clarity on the immune cell dynamics within the CNS. In the brains of infected mice, the expression levels of the pan-leukocyte marker CD45 were markedly elevated in CD45^+^ immune cells (Figures 5A and S3). This increase in CD45 fluorescence intensity underscored the immune activation triggered by CVB3 infection, although the total numbers of CD45^+^ cells remained similar. Microglia, identified by the cell surface expression of CD45, CD11b, and P2RY12, showed stable cell counts in infected brain tissue, indicating that - based on the identification by these markers - their numbers remained consistent during the infection (Figure 5B). However, microglial activation, particularly in terms of their antigen-presenting capacity, was evident, as indicated by the increased MHC class II levels (Figure 5C). Interestingly, the number of CD86-positive microglia, a co-stimulatory molecule indicative of improved antigen-presenting capacity, was rather reduced (Figure 5B). Furthermore, CD11c upregulation was observed in microglia following infection, consistent with previous findings in experimental models of autoimmune encephalomyelitis.^43^ Microglia responses in the infected mouse brain were also indicated by histological analysis of the brain, which showed an increase in mononuclear cells as well as Iba-1 positive microglia, particularly in the hippocampus of infected mice. Upon infection, microglia exhibited thicker processes and increased Iba-1 expression, both indicators of activation (Figure 5D). In addition to these phenotypical microglia responses, the strong induction of *Ccl2* mRNA expression (Figure 4G), known to be a potent monocyte chemoattractant, correlated with enhanced Ly6C^+^ monocyte infiltration (Figure 5E), while elevated *Cxcl10* and *Ccl5* mRNA expression (Figure 4G), known for attracting activated T cells, aligned with the increased T cell presence in the CNS, also confirmed by flow cytometry (Figure 5E). Histological analysis further validates T cell infiltration in the brain of individual mice, restricted to discrete focal regions but in close proximity to clusters of activated microglia (Figure S4). Importantly, there was no significant increase in B cells, dendritic cells, or neutrophils, underscoring a more selective recruitment of immune cells in response to CVB3 infection (Figure 5E). The immune responses, characterized by the induction of ISGs, cytokines, and chemokines, alongside extensive immune cell infiltration, were more pronounced in mice infected with the higher viral dose (1 × 10^6^ pfu), highlighting a dose-dependent activation of immune pathways during the acute phase of CVB3 infection. These findings emphasize the potent antiviral and inflammatory responses elicited in the CNS by CVB3 infection, with particular attention to microglial activation and the recruitment of immune cells.

### Persistent T cell presence and mild chronic immunopathology Post-CVB3 infection

**Figure 6:**
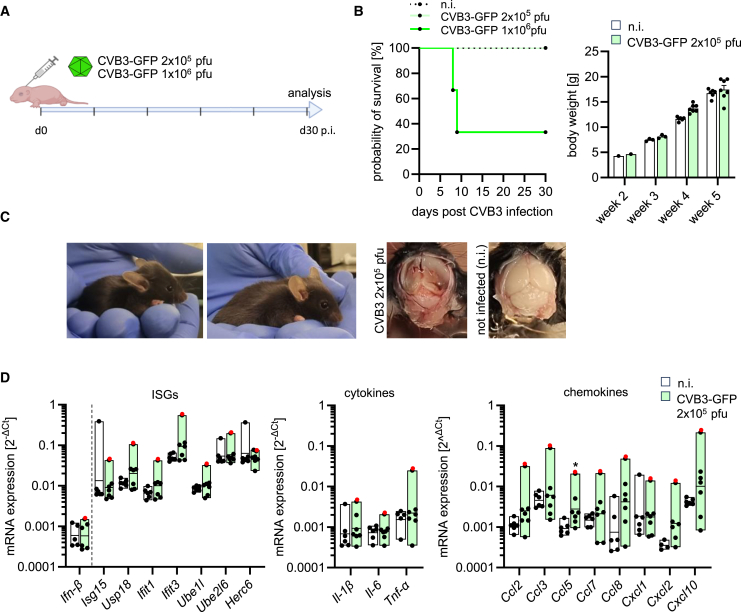
Mild chronic immunopathology following neonatal CVB3 infection (A) Neonatal mice were infected with 2 × 10^5^ or 1 × 10^6^ pfu CVB3-GFP (*n* = 7 per group) and analyzed on day 30 post-infection. (B) Mortality (left) and body weight changes (right) during the study. Initial infection with the higher dose (1 × 10^6^ pfu, *n* = 7) resulted in 70% mortality with some mice requiring euthanasia between days 7 and 9 post-infection due to ethical endpoints. (C) Left – Example of an infected mouse (2 × 10^5^ pfu) with hydrocephalus, compared to a mouse without visible abnormalities. Right – Brain of an infected mouse (2 × 10^5^ pfu) showing structural abnormalities, including cerebrum substance loss and enlarged ventricles, compared to a non-infected mouse. (D) mRNA expression of IFN-stimulated genes (ISGs), chemokines, and pro-inflammatory cytokines in the brains of infected (2 × 10^5^ pfu) and uninfected (n.i.) animals, quantified by qPCR (logarithmically transformed). Data were plotted as individual points, and results were presented as mean ± SEM. Statistical analysis by *t* test (*p* < 0.05). Significant results (*p* < 0.05) are marked with an asterisk (∗).

**Figure 7:**
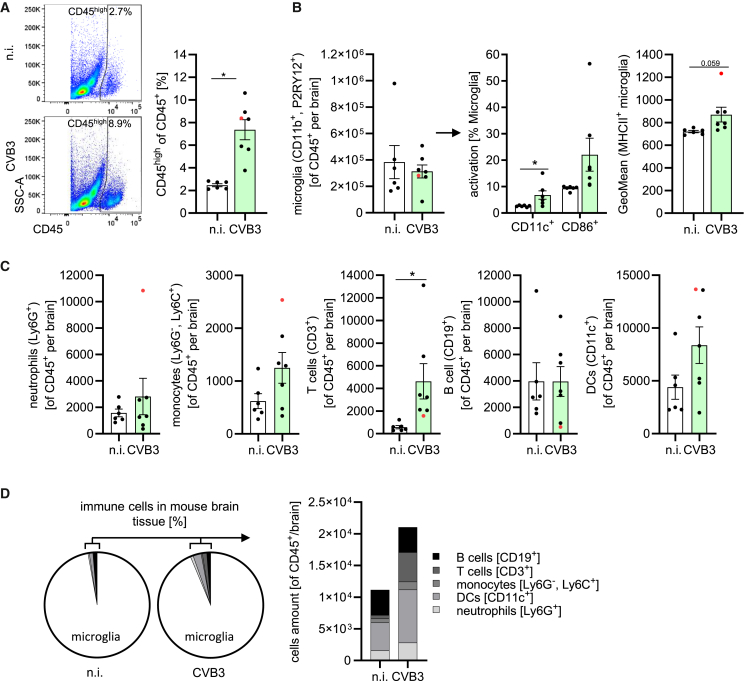
Ongoing low-grade immune cell infiltration with increased presence of T cells following neonatal CVB3 infection (A) Histogram (left) and quantitative analysis of CD45^high^ cells in the CD45^+^ population of neonatal mice infected with 2 × 105 CVB3 (*n* = 7) or uninfected mice (*n* = 6) analyzed on day 30 post-infection by flow cytometry (*t* test). Significant results (*p* < 0.05) are marked with an asterisk (∗). All data were plotted as individual points, and results were presented as mean ± SEM. (B) Left – Quantification of tissue-resident microglia (CD45^+^, CD11b^+^, P2RY12^+^); middle – analysis of activation markers by flow cytometry; right – geometric Mean fluorescence intensity (GeoMean) of MHC class II on microglia in infected and uninfected mice, determined by flow cytometry at day 30 post-infection. Statistical analysis by *t* test. Significant results (*p* < 0.05) are marked with an asterisk (∗). All data were plotted as individual points, and results were presented as mean ± SEM. (C) Quantitative analysis of immune cell presence in the brain by flow cytometry. Statistical analysis by *t* test. Significant results (*p* < 0.05) are marked with an asterisk (∗). All data were plotted as individual points, and results were presented as mean ± SEM. (D) Pie chart (left) outlines the proportional distribution of immune cells in the brains of infected and not infected mice, accompanied by a stacked bar chart (left) presenting the quantitative composition of inflammatory immune cell populations (shown as mean value).

The chronic phase following CVB3 infection was assessed at day 30 post-infection, a time point selected based on prior studies (Figure 6A).^20^ Initially, both viral doses (2 × 10^5^ pfu and 1 × 10^6^ pfu) were tested. However, significant lethality was observed in the higher-dose group, with 4 out of 7 pups succumbing to infection between days 7 and 9 post-infection. As a result, all subsequent analyses of the chronic phase were conducted using the lower dose (2 × 10^5^ pfu), where no mortality was observed (Figure 6B). Macroscopic inspections of the brain revealed hydrocephalus or softening of brain tissue in two animals, indicating post-infectious CNS abnormalities (Figure 6C). Notably, during the acute phase at the lower viral dose, minimal microglial activation and immune cell infiltration were observed. Therefore, a pronounced inflammatory response during the chronic phase was not anticipated. Likewise, IFN, cytokine, and chemokine responses were generally not elevated during this phase, with only occasional outliers showing minor immune activation. Notably, the most significant outlier (highlighted in red) was the mouse exhibiting a pronounced hydrocephalus and hindlimb weakness (Figure 6D). Consistent with the acute phase of CVB3 infection, flow cytometry showed elevated CD45 fluorescence intensity in CD45^+^ cells (Figure 7A). Microglia, defined as CD45^+^, CD11b^+^, and P2RY12^+^ cells, also displayed signs of continued activation (Figure 7B). Although their overall numbers remained stable, a subset of microglia exhibited upregulated CD11c expression, and markers, such as CD86 and MHC II, were modestly elevated, though these changes did not reach statistical significance (Figure 7B). Flow cytometry revealed significant T cell infiltration into the CNS during the chronic phase. Despite the absence of detectable viral RNA or infectious titers in the brain tissue (Figure S5), a substantial increase in T cell numbers was observed (Figure 7C). In contrast, the number of Ly6C^+^ monocytes and other immune cells remained unchanged (Figure 7C), while the stacked bar analysis pointed toward a slight increase in infiltrating immune cell populations in the brain of infected mice (Figure 7D). In conclusion, the chronic inflammatory phase did not trigger heightened IFN, cytokine, or chemokine responses. However, the unexpected and pronounced T cell infiltration, alongside ongoing microglial activation, suggests a persistent, low-level inflammatory process in the CNS, which may contribute to long-term neuroinflammatory pathology following CVB3 infection.

## Discussion

Enteroviruses are known for their ability to invade the central nervous system (CNS), causing a wide range of acute and chronic diseases. These can vary from mild conditions to severe, life-threatening illnesses, such as aseptic meningitis and encephalitis. These infections are particularly concerning in neonates, who are highly susceptible to viral infections. Once the virus enters the CNS, it triggers a complex immune response aimed at eliminating the pathogen and restoring balance. Microglia, the resident immune cells of the CNS, play a crucial role in this defense, acting as the brain’s primary immune surveillance system. Through pattern recognition receptors (PRRs), microglia detect viral pathogens and initiate a series of immune responses. While primary microglia cultures provide a controlled and accessible system to study microglial function, offering advantages such as reproducibility and mechanistic insights, they have inherent limitations, including altered gene expression profiles, loss of *in vivo* microenvironmental cues, and potential discrepancies in cellular behavior compared to resident microglia,^44^ necessitating complementary *in vivo* validation to ensure physiological relevance. This study investigates the responses in microglia upon CNS infection with CVB3, a model pathogen with neurotropism, in neonatal mice. We combined *in vivo* analysis of infected mice with cell culture data from primary microglia as well as primary neuronal cells, with neurons being the main cells infected by the virus.

Our *in vitro* experiments demonstrated that CVB3 replicates predominantly in neurons, while astrocytes and microglia remained uninfected, a result also reflected in *in vivo* studies. This confirms that neurons are the primary target cells for CVB3 in the CNS.^11^^,^^20^ The observed specificity could be attributed to neurons’ high expression of *Car*, which was undetectable in microglial cells (Figure S1D). While some studies have reported CVB3 replication in murine astrocytes,^45^ we did not detect this in our neuron-astrocyte co-cultures, at least not based on the immunofluorescent visualization of GFP, which is expressed during virus replication (Figure 1B). Despite strong viral replication observed in neurons, the induction of IFN responses and the production of chemokines and cytokines were relatively weak. This is in stark contrast to the robust immune activation seen with TLR agonists such as Poly(I:C) or Resiquimod, suggesting that neuronal cells possess the necessary machinery for a potent immune response but that CVB3 uses immune evasion mechanisms to suppress these pathways. Such immune evasion strategies are well-documented, particularly among picornaviruses.^46^ For example, CVB3 downregulates TLR3 and TRAF6, suppressing pro-inflammatory cytokine production through the NF-κB pathway.^47^ Additionally, CVB3 cleaves MAVS and TRIF, diminishing the host’s type I IFN response.^48^ These strategies likely explain the weak neuronal immune response observed in our studies. Notably, we detected neuronal production of *Ccl2*, which has also been observed in other picornavirus-induced CNS infections.^49^ In our acute infection model, there was a 110-fold elevated *Ccl2* mRNA expression, highlighting its role in recruiting immune cells.

Microglia are the primary immune responders in the CNS, and our *in vitro* data support their essential role during viral infections. CVB3 infection triggered the type I IFN pathway in microglia, leading to the expression of antiviral ISGs, such as *Ifit1, Ifit3*, and *Isg15* (Figure 2). Moreover, microglia induced ISGylation, a post-translational modification that strengthens antiviral defense by inhibiting viral replication.^50^^,^^51^^,^^52^ The most robust type I IFN response was induced by the TLR3 agonist Poly(I:C), showcasing microglia’s capacity to recognize viral RNA and mount a strong innate immune response.^53^ Furthermore, CVB3-stimulated microglia produced pro-inflammatory cytokines and chemokines, including *Il-6, Ccl5*, and *Cxcl10*, underlining their crucial role in orchestrating immune responses during viral infection.

*In vivo*, CVB3 infection in neonatal mice led to acute encephalitis, marked by a dose-dependent activation of type I IFN responses, cytokine production, microglial activation, and infiltration of peripheral immune cells. Contrary to previous studies, no virus-induced mortality was observed within the first five days post-infection, likely due to strain-specific differences in susceptibility combined with an efficient immune response.^10^ The robust type I IFN response, indicated by elevated *Ifn-β* mRNA levels, antiviral ISGs, and protein ISGylation in brain tissue, pointed to a highly effective antiviral defense in the CNS. Based on our *in vitro* data showing a limited IFN response in neuronal cells but potent signaling in primary microglia upon both CVB3 stimulation and TLR3/7 activation, we speculate that microglia are the primary contributors to the *in vivo* IFN response following CVB3 infection. Microglia quickly detect viral pathogen-associated molecular patterns (PAMPs) through PRRs, such as TLR3 and TLR7, responding within minutes of infection.^20^ While type I IFNs establish an antiviral state, microglia can also engage in phagocytosing CVB3-infected neurons.^20^ Our findings align with previous studies suggesting that microglia are not productively infected by CVB3 (Figures S1B and S1C). We hypothesize, based on previous findings with dendritic cells, that microglia are further activated by damage-associated molecular patterns (DAMPs) released by infected neurons.^54^^,^^55^ The immune response triggered by microglia includes various pro-inflammatory cytokines and chemokines, with *Tnf-α* being the most significantly upregulated (30-fold). TNF-α, along with IL-1β, modulates the integrity of the blood-brain barrier, facilitating immune cell infiltration.^56^ Chemokines, such as CCL2, CCL5, and CxCL10, play critical roles in recruiting immune cells to the CNS. Flow cytometry confirmed the infiltration of dendritic cells, Ly6C^+^ monocytes, and T cells during the acute phase.

In most cases, the acute immune response clears the virus from the CNS. However, under certain circumstances, viral RNA can persist for years, maintaining a low-level immune response that contributes to chronic neurological disorders.^57^^,^^58^ Specifically, we consider how persistent immune activation in our model might impact neuronal integrity and function. Although we did not detect viral RNA at day 30 post-infection, the persistent infiltration of T cells and continued microglial activation show ongoing low-level inflammation. Previous studies have demonstrated that in chronic flavivirus infections, sustained microglial cytokine expression and prolonged T cell activity can contribute to synapse elimination and cognitive dysfunction.^59^ Potential parallels between these findings and CVB3 infection may be drawn. Our observation of persisting microglia activation and elevated T cell presence is consistent with previous studies of persistent CVB3 RNA and chronic inflammation following neonatal infection.^20^ However, unlike prior studies in BALB/c mice infected with 2 × 10^6^ pfu of CVB3-GFP, we did not detect viral RNA at day 30 post-infection with 2 × 10^5^ pfu CVB3-GFP, suggesting that chronic immune activation can occur in the absence of viral persistence. The absence of viral RNA in our study may be due to infection with 10-fold less virus as well as mouse strain differences.^20^ BALB/c mice are highly susceptible to CVB3 infection, with 90% lethality within 10 days following intracerebroventricular infection with 2,5 × 10^5^ pfu,^10^ whereas C57BL/6 mice in our study showed 100% survival at a similar dose (Figure 6B). Additionally, BALB/c mice exhibit slower viral clearance compared to C57BL/6 mice following CVB3 infection^60^ which could further explain why viral RNA was detected in previous studies.^10^ Unexpectedly, our flow cytometry data revealed sustained T cell infiltration and continued microglial activation on day 30, indicating a chronic inflammatory process even without detectable viral RNA. This suggests that persistent low-level inflammation, rather than direct viral persistence, may drive prolonged microglial activation and immune infiltration. A similar phenomenon has been observed in chronic enterovirus infections in children, where sustained immune activation was linked to long-term neuroinflammatory pathologies.^61^ Additionally, the development of hydrocephalus in one mouse by day 30, as observed in our study, coincided with microglial overactivation, accompanied by increased ISG, cytokine, and chemokine expression. Together, these findings suggest that while CVB3 infection can trigger chronic neuroinflammation, the underlying mechanisms may depend on viral dose, host susceptibility, and immune response.^53^

Activation of the immune system, characterized by the expression of proinflammatory cytokines and chemokines and the infiltration of immune cells, appears to represent a common CNS response to both pathogens and tissue injury. Similar to our observations after CVB3 infection, stroke models such as MCAO (middle cerebral artery occlusion) show a comparable pattern of immune activation. In both models, DAMPs released from virus- or ischemia-induced cell damage and PAMPs from viral infection activate PRRs. While distinct PRRs are involved - TLR3 and TLR7 for CVB3-derived PAMPs, and TLR2, TLR4, and NOD-like receptors for MCAO-induced DAMPs - the downstream signaling induces type I interferons, proinflammatory cytokines (IL-1β, IL-6, and TNF-α), chemokines (CCL2, CCL3, and CXCL1), and interferon-stimulated genes (ISGs).^62^^,^^63^^,^^64^^,^^65^ The latter correlates with elevated ISGylation, observed in both ischemic and virus-infected brain tissue.^66^ Notably, ISGylation appears to be neuroprotective in stroke, but predominantly antiviral during infection.^50^ In the MCAO model, inflammation is marked by the early activation of microglia followed by the infiltration of T cells, monocytes, dendritic cells, and neutrophils.^63^^,^^67^^,^^68^^,^^69^ Except for neutrophils, a similar immune cell composition was detected in the CVB3-induced encephalitis model. MCAO studies further demonstrate accumulation and activation of microglia early after ischemia, with the expression of cytokines.^69^^,^^70^ Although microglial numbers did not increase in our study, FACS analysis and *in vitro* studies confirmed their activation and expression of *Il-1β*, *Tnf-α*, and *Il-6* after viral or TLR stimulation. Despite these similarities, the kinetics of the immune response differ markedly: the viral infection model displays a biphasic pattern with an acute antiviral phase followed by persistent T cell-mediated inflammation without detectable virus, whereas MCAO induces a typically monophasic inflammation determined by the initial tissue damage.

In conclusion, our study underscores the central role of microglia in defending the CNS against CVB3 infection. Activated by infected neurons, neuronal debris, and the virus itself, microglia initiate the type I IFN response and produce mediators that assist in recruiting peripheral immune cells, playing a key role in eliminating the virus. The sustained presence of T cells and prolonged microglial activation during the chronic phase suggests the potential for long-term neuroinflammatory consequences. Understanding how to modulate microglial activity to maintain effective antiviral defense while minimizing neuroinflammation will be critical for developing therapeutic strategies to mitigate the long-term effects of viral encephalitis.

### Limitations of the study

Microglia are pivotal in recognizing and combating neuronal pathogens, but other neuronal cells also play critical roles in maintaining immune homeostasis in the CNS. Beyond neurons, microglia interact with astrocytes through direct cell-cell contact and the release of soluble factors, forming a coordinated network to enhance the immune response. This interaction is essential not only for limiting viral replication but also for modulating inflammation to prevent excessive neural tissue damage. To deepen our understanding of the brain’s immune response to CVB3 infection, future studies should include investigations of astrocytes’ contributions to this process.

## Resource availability

### Lead contact

Further information and requests for resources and reagents should be directed to and will be fulfilled by the Lead Contact, Prof. Antje Beling (antje.beling@charite.de).

ORCID ID: 0000-0002-1826-5248.

### Materials availability

This study did not generate new unique reagents.

### Data and code availability

• Data: All data reported in this article will be shared by the lead contact upon request.
• Code: This article does not report original code.
• Additional information: Any additional information required to reanalyze the data reported in this article is available from the lead contact upon request.

## Acknowledgments

We acknowledge Karolin Voss and Prisca Kunert for excellent technical assistance and the technical support of the BIH Cytometry Core Facility. The Advanced Medical Bioimaging Core Facility at the Charité supported this project with confocal microscopy. A.B. received support from the Foundation for Experimental Biomedicine, Zurich, Switzerland (2016–2023). The project was funded by the 10.13039/501100001659Deutsche Forschungsgemeinschaft (DFG, 10.13039/501100001659German Research Foundation, Germany) – projects: 318346496 – CRC 1292 (TP02); 259373024 – TRR 167 (B16); 437531118 – CRC 1470 (A08); 254158365 to A.B.. M.S. is supported by the Berta-Ottenstein-Programme for Clinician Scientists, 10.13039/501100021729Faculty of Medicine, University of Freiburg, Germany, and the IMM-PACT-Programme for Clinician Scientists, Department of Medicine II, Medical Center – 10.13039/501100002714University of Freiburg and Faculty of Medicine, University of Freiburg, Germany, funded by the Deutsche Forschungsgemeinschaft (DFG, German Research Foundation, Germany) – 413517907. Light microscopy imaging was performed at the Lighthouse Core Facility, which is funded in part by the 10.13039/501100021729Medical Faculty, University of Freiburg, Germany (Project Numbers 2023/A2-Fol; 2021/B3-Fol), the 10.13039/501100012353DKTK, Germany, and the DFG (Project Number 450392965). L.M.S. received a doctoral scholarship from CRC/TRR 167 NeuroMac as part of the graduate program IRTG (NeuroMac School).

## Author contributions

Conceptualization, A.B. and S.P.; methodology, S.P., L.M.S., A.B., L.G.M.H., T.S., M.J., and M.O.; investigations, S.P., L.M.S., L.G.M.H., M.S., M.J., and M.O.; formal analysis, S.P., L.M.S., M.J., and M.O.; writing – original draft, L.M.S., S.P., and A.B.; writing – review and editing, L.M.S., S.P., A.B., L.G.M.H., M.S., and M.J.; visualization, L.M.S., S.P., and M.S.; project administration, A.B. and S.P.; funding acquisition, A.B.

## Declaration of interests

The authors declare no competing interests.

## Declaration of generative AI and AI-assisted technologies in the writing process

This article was prepared with the assistance of ChatGPT, a language model developed by OpenAI, which was used for refining language. The authors have reviewed, revised, and approved the final content.

## STAR★Methods

### Key resources table

**Table:** 

REAGENT or RESOURCE	SOURCE	IDENTIFIER
**Antibodies**		

CD3ϵ (BUV737)	BD Bioscience	clone 145-2C11; Cat.#612771
I-[Ab] (BV421)	BD Bioscience	clone AF6-120.1; Cat.#566229
CD19 (BV605)	BD Bioscience	clone 1D3; Cat.#563058
Ly-6G (BV786)	BD Bioscience	clone 1A8; Cat.#569406
CD11c (BB700)	BD Bioscience	clone HL3; Cat.#566504
Ly-6C (PE-CF594)	BD Bioscience	clone AL-21; Cat.#562728
CD45.2 (BV711)	BD Bioscience	clone 104; Cat.#563685
F4/80 (R718)	BD Bioscience	clone T45-2342; Cat.#752152
CD86 (BUV395)	BD Bioscience	clone GL1; Cat.#564199
P2RY12 (PE)	BioLegend	clone S16007D; Cat.#848003
CD11b (PE-Cy7)	eBioscience	clone M1/70; Cat.#25-0112-81
Tubulin	Merck	clone DM1A; Cat.#T9026
Actin	Merck	clone C4; Cat.#MAB1501
mISG15	Dr. Klaus-Peter Knobeloch, Freiburg, Germany	N/A
mUSP18	Invitrogen	Cat.#PA5-110555
STAT1	Cell Signaling Technology	Cat.#9172S
pSTAT1	Cell Signaling Technology	Cat.#9167S
VP1	Mediagnost	clone 31A2; Cat.#M47
goat anti mouse IRDye^TM^ 800CW	LI-COR	Cat.#926-32210
goat anti mouse IRDye^TM^ 680RD	LI-COR	Cat.#926-68070
goat anti rabbit IRDye^TM^ 800CW	LI-COR	Cat.#926-32211
goat anti rabbit IRDye^TM^ 680RD	LI-COR	Cat.#926-68071
mouse anti-Map2	Sigma Aldrich	Cat.#M9942
guinea pig anti-GFAP	Synaptic Systems	Cat.#173004
anti-guinea pig Cy3	Dianova	Cat.# 706-165-148
anti-mouse Alexa Fluor 647	Thermo Fisher Scientific	Cat.#A-21235
Iba1	Abcam	Cat.#178846
goat anti-rabbit IgG	SouthernBiotech	Cat.#4050-08

**Recombinant DNA**		

pMKS1	Dr. Henry Fechner, Berlin; Germany	N/A
pMKS1-eGFP	Dr. Henry Fechner, Berlin; Germany	N/A

**Chemicals, peptides, and recombinant proteins**		

PEI (polyethylenimine) Max	Polysciences	Cat.#24765
BD Difco Agar Noble	Thermo Fisher Scientific	Cat.#11798223
cOmplete Protease Inhibitor Cocktail	Roche	Cat.#11697498001
PhosphoSTOP phosphatase inhibitor	Roche	Cat.#4906845001
MTT (3-/4,5-dimethylthiazol-2-yl)-2,5-diphenyltetrazolium bromide)	Sigma Aldrich	Cat.#M2128
Poly-L-Lysine	Merck	Cat.#P7890
mouse GM-CSF	Miltenyi Biotec	Cat.#130-095-746
M-CSF	PeproTech	Cat.#315-02
Neurobasal medium	Gibco	Cat.#21103049
B-27™	Gibco	Cat.#17504044
DNase I	Sigma Aldrich	Cat.#04716728001
Poly-DL-Ornithine	Merck	Cat.#P8638
GlutaMAX	Gibco	Cat.#35050061
Horse serum	Gibco	Cat.#26050088
Poly(I:C)	InvivoGen	Cat.#tlrl-pic
Resiquimod	Stemcell Technologies	Cat.#73782
murine Interferon-β	Sigma Aldrich	Cat.#I9032-1VL
MLV Reverse Transcriptase	Promega	Cat.#M1705
random hexamer primers	Roche	Cat.#11034731001
pHrodo™ Red Zymosan BioParticles™	Thermo Fisher Scientific	Cat.#P35364
Cytochalasin D	Sigma Aldrich	Cat.#C8273
streptavidin peroxidase	SouthernBiotech	Cat.#7105-05
normal goat serum	SouthernBiotech	Cat.#0060-01

**Critical commercial assays**		

DAB substrate kit	Abcam	Cat.#64238
TRIzol	Thermo Fisher Scientific	Cat.#15596026
TaqMan gene expression assays	Thermo Fisher Scientific	Cat.#4331182
interferon beta 1	Thermo Fisher Scientific	Assay ID: Mm00439546_s1
USP18	Thermo Fisher Scientific	Assay ID: Mm01188805_m1
ISG15	Thermo Fisher Scientific	Assay ID: Mm01705338_s1
Ube1L	Thermo Fisher Scientific	Assay ID: Mm00612563_m1
Ube2l6	Thermo Fisher Scientific	Assay ID: Mm00498295_m1
Herc6	Thermo Fisher Scientific	Assay ID: Mm01341950_m1
mIFIT1	Thermo Fisher Scientific	Assay ID: Mm00515153_m1
mIFIT3	Thermo Fisher Scientific	Assay ID: Mm01704846_s1
interleukin 6	Thermo Fisher Scientific	Assay ID: Mm99999064_m1
interleukin 1 beta	Thermo Fisher Scientific	Assay ID: Mm00434228_m1
TNF-alpha	Thermo Fisher Scientific	Assay ID: Mm99999068_m1
Ccl2	Thermo Fisher Scientific	Assay ID: Mm00441242_m1
Ccl3	Thermo Fisher Scientific	Assay ID: Mm00441259_g1
Ccl5	Thermo Fisher Scientific	Assay ID: Mm01302427_m1
Ccl7	Thermo Fisher Scientific	Assay ID: Mm00443113_m1
Ccl8	Thermo Fisher Scientific	Assay ID: Mm01297183_m1
Cxcl1	Thermo Fisher Scientific	Assay ID: Mm04207460_m1
Cxcl2	Thermo Fisher Scientific	Assay ID: Mm00436450_m1
Cxcl10	Thermo Fisher Scientific	Assay ID: Mm00445235_m1

**Experimental models: cell lines**		

HEK293T cells	ATCC	N/A
HeLa cells	ATCC	ATCC CCL-2

**Experimental models: organisms/strains**		

C57BL/6J	Animal facility Charitè	N/A

**Oligonucleotides**		

murine HPRT; forward primer 5′-ATC ATT ATG CCG AGG ATT TGG AA-3′	Our lab	N/A
murine HPRT; reverse primer 5′-TTG AGC ACA CAG AGG GCC A-3′	Our lab	N/A
murine HPRT; probe 5′FAM-TGG ACA GGA CTG AAA GAC TTG CTC GAG ATG-3′ TAMRA	Our lab	N/A
CVB3; forward primer 5′-CCC TGA ATG CGG CTA ATC C-3′	Our lab	N/A
CVB3; revers primer 5′-ATT GTC ACC ATA AGC AGC CA-3′	Our lab	N/A
CVB3; probe 5′-FAM-TGC AGC GGA ACC G-MGB3′	Our lab	N/A

**Software and algorithms**		

Image Studio Lite software version 5.2	LI-COR biotechnology	Image Studio 5.2 Download - iidf.exe
FlowJo v10.9.0	Ashland	FlowJo - Download | FlowJo, LLC
GraphPad Prism v10.00	GraphPad Software	GraphPad Prism 10 User Guide - Installing Prism

**Other**		

StepOnePlus real-time PCR system	Thermo Fisher Scientific	Cat.#15320855
Pneumatic Picopump	World Precision Instruments	Cat.#PV830
glass cannulas	Kimble melting point capillary	Cat.#KIM3450599
Pip 6 pipette puller	HEKA	N/A
Odyssey CLx infrared system	LI-COR biotechnology	N/A
Nikon Scanning, Confocal A1Rsi+	Nikon Europe BV	N/A
Zeiss Axioscan 7 system	Zeiss	N/A
FACS Symphony flow cytometer	BD Biosciences	N/A

### Experimental model and study participant details

#### Mice *in vivo* experiments

For *in vivo* studies, C57BL/6J pups were used. Animals were maintained under standard housing conditions in compliance with the regulations of EU Directive 2010/63/EU and Commission Recommendation 2007/526/EC. Housing conditions included unrestricted access to food and drinking water (*ad libitum*), a 12-hour light/dark cycle, appropriate bedding and nesting material, shelters, and cage enrichment. The hygiene management concept ensures strict separation between the central breeding facility and the experimental units. The maintenance of a specified pathogen-free (SPF) health status is regularly monitored - four times per year in the breeding facility and twice per year in the experimental units.

For the experiments, pregnant dams were transported to the experimental facility one week prior to the expected delivery date to allow acclimatization and habituation to the researcher. Neonates were used for experiments within 24 hours after birth. To prevent viral contamination of the uninfected controls, all littermates of a given litter were assigned either to the infection or control group and were housed together with their respective dam. During the time of the experiment, pups stayed together with their litter mates and mothers to ensure adequate nursing and care. To minimize suffering, Tramadol (Grünenthal, Stolberg, Germany) 0.1 mg/ml was administered orally via drinking water. Water was renewed daily, and treatment with Tramadol began seven days prior to infection and was continued until the final day of the experiment.^71^ Additionally, to normal standard food, wet food was offered to the mice from day 10 until end of the experiment. Mice were monitored daily.

Sex differences in disease susceptibility are well-established in virus infection, in particularly CVB3 infection. However, sex differentiation of the neonates was not performed, as sex cannot be reliably determined within the first 24 hours after birth. Therefore, both male and female mice were included in the experiments.

All animal experiments were conducted in accordance with the German Animal Welfare Act and the European Directive 2010/63/EU on the protection of animals used for scientific purposes. The study was approved by the local animal welfare authorities in Berlin (Landesamt für Gesundheit und Soziales), registered under permit number G0009/22. Animals that met termination criteria outlined in the score sheets (no visible milk belly or isolation from the group for longer than 12 hours, reduced motion and bluish/greyish skin color for more than 48 hours) were humanely euthanized to prevent undue suffering.

#### Primary cultures

For primary microglia, brains from C57BL/6J pups (P1-P3, mixed sex) were collected in HBSS (Hanks’ Balanced Salt Solution; Thermo Fisher Scientific). The cerebellum and olfactory bulbs were removed, and hemispheres were rolled over Whatman paper (GE Healthcare, Chicago, IL/USA) to strip the meninges. Tissue was mechanically dissociated and digested with 0.01% Trypsin (Gibco, Life Technologies, Carlsbad, CA/USA) at 37°C for 12-15 min. Microglia medium (DMEM (Gibco, Life Technologies) with 10% FCS, 1% penicillin/streptomycin (Gibco, Life Technologies), and 1 mM Sodium Pyruvate (Gibco, Thermo Fisher Scientific) was added, and cells were centrifuged at 300 x g for 8 min. The cell pellet was resuspended in microglia medium and transferred to Poly-L-Lysine (20 μg/ml, Merck, Sigma Aldrich, Darmstadt, Germany)-coated T75 flasks (2 brains/flask). After 5 days, the medium was replaced with fresh microglia medium, and on day 8, supplemented with 10 ng/ml mouse GM-CSF (Miltenyi Biotec, Bergisch Gladbach, Germany). Cells were harvested on days 12 or 13.

Adult microglia cultures were obtained from brain tissues of 6 to 10 weeks old C57BL/6J mice (mixed sex) as described previously. The brains were isolated after transcardiac perfusion with 20 ml PBS after an overdose of isoflurane. The brain was isolated and transferred into a glass potter filled with 10 mL cold dissection medium (HBSS, 25 mM HEPES, 1.3% Glucose (45%, Sigma Aldrich)). After homogenization and transfer through a 70 μm cell strainer (Corning Inc., Corning, NY/USA), cells were centrifuged (10 min at 400 x g, 4°C) and cell pellet resuspended in 10 ml of 37% Percoll/PBS (Cytiva, Wilmington, DE/USA). After centrifugation (30 min at 1000 x g and 4°C without brake) the cell pellet was additionally washed once with 10 mL PBS and then again centrifuged at 300 x g for 5 min at 4°C. The cell pellet was resuspended in 10 mL adult microglia medium (DMEM, 4.5 g/l Glucose (Gibco, Thermo Fisher Scientific), 10% heat inactivated FCS, 1% penicillin/streptomycin, 20 ng/ml M-CSF (PeproTech, Thermo Fisher Scientific), 20 ng/ml IL-34 (BioLegend, San Diego, CA/USA). The cell suspension was then plated into previously PEI-coated T25 flasks. Three brains were cultured per flask and cells were cultured at 3% oxygen, with a partial medium change at day 2 and 7. On day 14 after preparation, adult microglia cells were harvested by Trypsin/EDTA (0.05%), plated in 24-well plates and stimulated as described. The used isolation protocol based on the method described by Aktories et al., 2022.^72^ Purity of both microglia cultures was regularly checked by flow cytometry using CD45 and CD11b specific antibodies.

For primary neuronal cells, embryos (E16, mixed sex) from C57BL/6J mice were collected in ice-cold PBS. Brains were removed and dissected to remove the cerebellum and olfactory bulbs. Meninges were carefully removed under a microscope, and cortical hemispheres were digested with 1 mg/ml Trypsin (Worthington, Lakewood, NJ/USA) for 10 min at 37°C. Following digestion, tissue was washed in HBSS, and resuspended in Neurobasal medium (Gibco, Thermo Fisher Scientific) supplemented with 10% horse serum (Gibco, Thermo Fisher Scientific). Cells were centrifuged and resuspended in Neurobasal-complete medium containing 2% B-27™ (Gibco, Thermo Fisher Scientific), 1% GlutaMAX (Gibco, Thermo Fisher Scientific), 1% penicillin/streptomycin, and 0.072% 2-Mercaptoethanol (Gibco, Thermo Fisher Scientific), with DNase I (0.15 mg/ml; Sigma Aldrich) added. Cells were passed through a 100 μm filter (Greiner Bio-One) and plated in Poly-DL-Ornithine (1.5 mg/ml, Merck, Sigma Aldrich) -coated 12-well plates. Neurons were cultured in Neurobasal-complete medium for 7 days to grow before using them for experiments. HeLa cells (ATCC CCL-2) were cultured in MEM (Life Technologies) supplemented with 5% FCS and 1% penicillin/streptomycin and incubated at 37°C. HEK 293T cells were cultured in high-glucose DMEM (Gibco, Thermo Fisher Scientific) supplemented with 10% FCS and 1% penicillin/streptomycin.

### Method details

#### Virus propagation and quantification

Viruses were propagated by transfection of cDNA plasmids of Coxsackievirus B3 H3 strain with or without eGFP (CVB3-GFP)^73^ into HEK293T cells at a confluence of 80% using PEI (polyethylenimine) Max (Polysciences, Warrington, PA, USA) and amplified once in HeLa cells. The virus for *in vivo* application was purified and concentrated by ultracentrifugation through a 30% sucrose solution before use. The amount of infectious virus after propagation as well as viral titers from *in vitro* and *in vivo* experiments were determined by plaques assay using HeLa cells. Therefore, confluent HeLa cells were incubated with 10-fold dilutions of virus solution, cell culture supernatants or homogenized organs for 30 min at 37°C. After incubation, the supernatants were discarded carefully and HeLa cells were overlaid with Eagle’s-agar (MEM, 1% penicillin/streptomycin, 3.2 g/L NaHCO_3_, 9% FCS (fetal calf serum, Sigma Aldrich, St. Louis, MO/USA) and 0.7% BD Difco Agar Noble (Thermo Fisher Scientific, Waltham, MA/USA)). Cells were stained with MTT (3-/4,5-dimethylthiazol-2-yl)-2,5-diphenyltetrazolium bromide; Sigma Aldrich), two to three days after infection, incubated for up to four hours, and plaques were counted subsequently.

#### Intracerebroventricular (i.c.) injection of mice

C57BL/6J pups (less than 24 h old) were injected into the cerebral ventricles with 2 μl of virus solution containing CVB3-GFP (1x10^6^ pfu or 2x10^5^ pfu, 0.1% Fast Green (Sigma Aldrich) in 0.9% NaCl), or 2 μl of control solution (0.1% Fast Green in 0.9% NaCl). Pups were anesthetized via hypothermia and injected in the lateral ventricle at the lambda line (from Lambda: 1 mm rostral, 1 mm lateral, 1.5 mm ventral)^74^ using a Pneumatic Picopump (World Precision Instruments, Sarasota, Florida USA). Before injection, glass cannulas (Kimble melting point capillary, DWK Life Sciences, New Jersey, USA) were prepared using the Pip 6 pipette puller (HEKA, temperature-controlled pipette puller, Massachusetts USA) according to the manufacturer’s instructions. Pups were monitored for recovery and returned to their mothers. Injections were monitored twice on the day of injection and daily thereafter. At 5 days post-injection, pups were sacrificed by decapitation. Mice at 30 days post-injection were anesthetized using 100 mg/kg Ketamin (Essex Tierarznei, München, Germany) and 30 mg/kg Xylazine/Rompun (Bayer, Leverkusen, Germany) diluted with 0,9% NaCl and perfused with cold PBS (Gibco, Thermo Fisher Scientific). The heart, spleen, liver, pancreas, and brain were collected. The organs were snap-frozen in liquid nitrogen and stored at -80°C for further analysis. Tissues for immunohistochemistry were fixed in 4% formaldehyde solution (Carl Roth, Karlsruhe, Germany) and paraffin embedded.

#### Stimulation of cells for qPCR and immunoblotting

Cells were plated in 6- or 12-well plates. After 24 h, cells were stimulated with Poly(I:C) 50 μg/ml (InvivoGen, San Diego, CA/USA), Resiquimod 10 μg/ml (Stemcell Technologies, Vancouver, Canada), murine Interferon-β (IFN-β, Sigma Aldrich) 100 U/ml, or CVB3 MOI 0.5, 5, or 50 for up to 24 h. RNA samples were harvested 24 h after stimulation, while protein samples for immunoblotting were collected at 0, 0.5, 8, and 24 h. Cells were scraped, centrifuged, and lysed in either CHAPS-based lysis buffer (1% CHAPS (Thermo Fisher Scientific), 20 mM HEPES (AppliChem, Darmstadt, Germany), 8 mM EDTA (Boehringer, Ingelheim, Germany), 2 mM EGTA (Boehringer), 50 mM NaF (Thermo Fisher Scientific), 5 mM NaPP (Thermo Fisher Scientific), 2 mM Na_3_VO_4_ (Thermo Fisher Scientific), 10 mM NEM (Sigma Aldrich), 2X cOmplete Protease Inhibitor Cocktail (Roche, Basel, Switzerland)) or PhosphoSTOP-based lysis buffer (2X PhosphoSTOP phosphatase inhibitor (Roche), 100 mM Tris-HCl pH 7.5 (AppliChem), 10% glycerol (AppliChem), 8 mM EDTA, 2 mM EGTA, 2 mM NEM, 2 mM TCEP (tris(2-carboxyethyl)phosphine; Merck, Sigma Aldrich), 2% Triton X-100 (ACROS Organics, Thermo Fisher Scientific), 2X cOmplete Protease Inhibitor Cocktail), snap-frozen and stored at -80°C. PhosphoSTOP-based lysis buffer was used for all immunoblots that investigated phosphorylation events. Otherwise, CHAPS-based lysis buffer was used.

#### RNA isolation and quantitative real-time PCR (qPCR)

RNA was extracted using TRIzol (Thermo Fisher Scientific) following the manufacturer’s instructions. The final RNA concentration was measured using a NanoDrop spectrophotometer (VWR, Radnor, PA/USA). 250 – 1000 ng of RNA was reverse transcribed using MLV Reverse Transcriptase (Promega, Madison, WI/USA) with random hexamer primers (Roche). TaqMan PCR was performed with specific primers and probes using TaqMan gene expression assays (Life Technologies, Thermo Fisher Scientific) as well as the following combinations of primers and probes: murine HPRT; forward primer 5′-ATC ATT ATG CCG AGG ATT TGG AA-3′, reverse primer 5′-TTG AGC ACA CAG AGG GCC A-3′ and probe 5′FAM-TGG ACA GGA CTG AAA GAC TTG CTC GAG ATG-3′ TAMRA, and CVB3; forward primer 5′-CCC TGA ATG CGG CTA ATC C-3′, revers primer 5′-ATT GTC ACC ATA AGC AGC CA-3′ and probe 5′-FAM-TGC AGC GGA ACC G-MGB3′. Reactions were run on the StepOnePlus real-time PCR system (Thermo Fisher Scientific). HPRT (hypoxanthinguaninphosphoribosyltransferase) was used as an endogenous control for relative quantification using the ΔC(t) or ΔΔC(t) method.

#### Protein quantification and immunoblotting

Protein concentration was measured using Bradford assay (Thermo Fisher Scientific) according to manufacturer’s guidelines. Lysates were denatured and loaded onto SDS-PAGE gels for separation, followed by transfer to PVDF membranes (Odyssey Nitrocellulose Membranes, LI-COR biotechnology, Lincoln, NE/USA). Blots were incubated with primary antibodies, washed, and exposed to secondary antibodies. Proteins were visualized using an Odyssey CLx infrared system (LI-COR biotechnology). Densitometry was performed using Image Studio Lite software version 5.2 (LI-COR biotechnology).

The following primary antibodies were used: tubulin (Merck, Sigma Aldrich, dilution 1:5,000, clone DM1A), actin (Merck, Millipore, Billerica, MA/USA, dilution 1:5,000, clone C4), mISG15 (Klaus-Peter Knobeloch, Freiburg, Germany, dilution 1:1,000), mUSP18 (Invitrogen, Thermo Fisher Scientific, dilution 1:1,000), STAT1 (Cell Signaling Technology, Danvers, MA/USA, dilution 1:1,000), pSTAT1 (Cell Signaling Technology, dilution 1:1,000), VP1 (Mediagnost, Reutlingen, Germany, dilution 1:2,000, clone 31A2). The following secondary antibodies were used: goat anti mouse IRDye^TM^ 800CW (LI-COR, dilution 1:10,000), goat anti mouse IRDye^TM^ 680LT (LI-COR, dilution 1:20,000), goat anti rabbit IRDye^TM^ 800CW (LI-COR, dilution 1:10,000), goat anti rabbit IRDye^TM^ 680LT (LI-COR, dilution 1:20,000).

#### Immunofluorescence

For immunofluorescence microscopy neuronal cultures were seeded on 13-mm glass coverslips coated with Poly-DL-Ornithine. Day 14 after preparation, cells were washed with PBS and fixed with 4% formaldehyde solution (Carl Roth) for 20 min at RT. Cell were permeabilized with 0.2% Triton X-100 (Sigma Aldrich) for 10 min, after additional wash step with PBS. To avoid non-specific binding, cells were blocked with 4% FCS/PBS/0.1% Triton X-100 for 30 min. Incubation with primary antibody mouse anti-Map2 (Sigma Aldrich, dilution 1:500) or guinea pig anti-GFAP (Synaptic Systems, dilution 1:400, Goettingen; Germany) was performed in blocking buffer for 1 h at RT. After three washing steps with PBS, samples were incubated with secondary antibody anti-guinea pig Cy3 (Thermo Fisher Scientific, dilution 1:300) or anti-mouse Alexa Fluor 647 (Thermo Fisher Scientific, dilution 1:200), in blocking buffer at RT for 1 h. After three washing steps with PBS, samples were mounted in ROTI-Mount Fluor-Care (Carl Roth). Images were acquired with a Nikon Scanning, Confocal A1Rsi+ (Nikon Europe BV, Amsterdam, Netherlands).

#### Immunohistochemistry

Chromogenic immunohistochemistry was performed on 3 μm–thick FFPE tissue sections. Briefly, slides were incubated at 80°C for 1 h to initiate deparaffinization, followed by immersion in Xylene (Sigma Aldrich) for complete paraffin removal. Endogenous tissue peroxidase activity was blocked using 3% hydrogen peroxide (merck) for 10 min. Non-specific binding was blocked with 10% normal goat serum (SouthernBiotech, Birmingham, AL, USA) containing 1% Triton X-100 in TRIS buffer (EnVision Flex Wash Buffer, Agilent, Santa Clara, CA, USA) for 1 h. Sections were then incubated overnight at 4°C with a primary anti-Iba1 antibody (Abcam, Cambridge, England 1:1000) diluted in 10% normal goat serum and 1% Triton X-100 in TRIS buffer. After three washing steps in TRIS buffer, slides were incubated with a goat anti-rabbit secondary antibody (Southern Bioscience, 1:300) in 10% normal goat serum, 1% Triton X-100 in TRIS buffer for 45 min at room temperature. Following another three washes, sections were treated with 0.1% streptavidin–peroxidase (SouthernBiotech) in TRIS buffer for 45 min at room temperature and washed three additional times. Next, slides were incubated with DAB solution (Abcam), with staining progress continuously monitored using a light microscope to determine optimal incubation time. Slides were counterstained with Gill’s hematoxylin (Sigma Aldrich). Vitro-Clud (R. Langenbrinck GmbH, Emmendingen, Germany) was used as mounting medium. Imaging was conducted using a Zeiss Axioscan 7 microscope equipped with a 20× objective.

#### MTT assay for cell viability

Neurons were plated in 96-well plates and stimulated with Poly(I:C) (50 μg/ml), Resiquimod (10 μg/ml), IFN-β (100 U/ml), or CVB3-GFP (MOI 0.5). After 24 hours, MTT (3-(4,5-dimethyl-thiazol-2-yl)-2,5-diphenyltetrazolium bromide; Sigma Aldrich) was added to a final concentration of 0.5 mg/ml and incubated for 3 hours. Cells were lysed with isopropanol (AppliChem), and absorbance was measured with a plate reader Synergy HT, Bradford measurement (BioTek, Winooski, VT/USA) at 570 nm.

#### Phagocytosis assay

Microglia were stimulated with Poly(I:C) (50 μg/ml), Resiquimod (10 μg/ml), or CVB3 (MOI 50) for 24 hours. Cells were incubated with pHrodo™ Red Zymosan BioParticles™ (0.5 mg/ml; Invitrogen, Thermo Fisher Scientific) according to manufacturer’s instructions, and fluorescence was measured using a plate reader (Ex: 520 nm, Em: 580 – 640 nm, GloMax Discover, Promega). Cytochalasin D (10 μM, Sigma Aldrich) was used to block phagocytosis as negative control.

#### Isolation of immune cells from mouse tissue and flow cytometry

Mice were sacrificed, and brains were harvested. The cerebellum was removed and discarded. The remaining brain tissue was stored in ice-cold dissection medium (HBSS, 15 mM HEPES (Life Technologies), 30 mM d-Glucose (Sigma Aldrich) per brain. Brains were minced into small pieces and passed through a 70 μm cell strainer (Corning Inc.). Fresh dissection medium was added, and the cell suspension was centrifuged at 800 x g for 5 min at 4°C. After discarding the supernatant, the cell pellet was resuspended in 10 ml of 37% Percoll (Cytiva) solution diluted in PBS and centrifuged without applying a brake at 800 x g for 30 min at 4°C. The myelin layer and supernatant were carefully removed, and the cell pellet was resuspended in FACS buffer (PBS with 2% FCS (Sigma Aldrich) and 2 mM EDTA (Ethylenediaminetetraacetic acid, VWR)). Cells were centrifuged again at 800 x g for 5 min at 4°C, the supernatant was discarded, and the cell pellet was resuspended in FACS buffer. For single stains, splenocytes were used. For splenocyte isolation, spleen tissue was passed through a 100 μm cell strainer, followed by red blood cell lysis. Therefore, the cell pellet was re-suspended in 0.83% ammonium chloride (NH_4_Cl) in PBS. The solution was incubated for three minutes at room temperature and centrifuged afterwards to recover the cells. The splenocytes were re-suspended in FACS buffer and stored on ice until antibody incubation. Equal numbers of immune cells were incubated for 20 min at 4°C with FcR blocking reagent (Miltenyi Biotec) diluted 1:50 in FACS buffer. Fluorochrome-conjugated antibodies were added and incubated for 20 min at 4°C. The following antibodies were used: CD3ϵ (BUV737; clone 145-2C11, 1:200), I-[Ab] (BV421; clone AF6-120.1, 1:100), CD19 (BV605; clone 1D3, 1:100), Ly-6G (BV786; clone 1A8, 1:100), CD11c (BB700; clone HL3, 1:100), Ly-6C (PE-CF594; clone AL-21, 1:200), CD45.2 (BV711; clone 104, 1:200), F4/80 (R718; clone T45-2442, 1:400), CD86 (BUV395; clone GL1, 1:200) from BD Bioscience, San Jose, CA/USA; P2RY12 (PE; clone S16007D, 1:200) from BioLegend and CD11b (PE-Cy7; clone M1/70, 1:200) from eBioscience (San Diego, CA/USA). After washing, cells were resuspended in fixable viability dye eFluor 780 (eBioscience), diluted 1:1,000 in PBS, and incubated for 30 min at 4°C. Cells were washed with PBS and fixed with 2% HistoFix (Carl Roth) in PBS, then washed and resuspended in FACS buffer. Data were acquired using a FACS Symphony flow cytometer (BD Biosciences) and analyzed using FlowJo v10.9.0 software (Ashland, Wilmington, DE, USA).

### Quantification and statistical analysis

#### Statistics

Statistical analysis was performed using GraphPad Prism v10.00. Data were plotted as individual points, and results were presented as mean ± SEM. Unpaired t-tests were used for two-group comparisons, while unequal variance ANOVA (one-way or two-way) was used for multiple comparisons. A significant threshold of *p* < 0.05 was applied to all tests. The exact value of n and the statistical tests used are indicated in the respective figure legends.
