# Supplementary material for: Decoding IFN-mediated immunity and cell dynamics in viral encephalitis: Insights from coxsackievirus B3 infection
Source: iScience. 2025 Dec 3;29(1):114324. doi: 10.1016/j.isci.2025.114324 (PMC12768882; doi:10.1016/j.isci.2025.114324)
Supplement: Document S1. Figures S1–S6 [file mmc1.pdf]

iScience, Volume *29*

Supplemental information

Decoding IFN-mediated immunity

and cell dynamics in viral encephalitis:

Insights from coxsackievirus B3 infection

Lisa Marie Stach, Lisa Gerarda Maria Huis in `t Veld, Theres Schaub, Marina Jendrach, Marta Ornaghi, Marius Schwabenland, Antje Beling, and Sandra Pinkert

Supplemental Figure S1

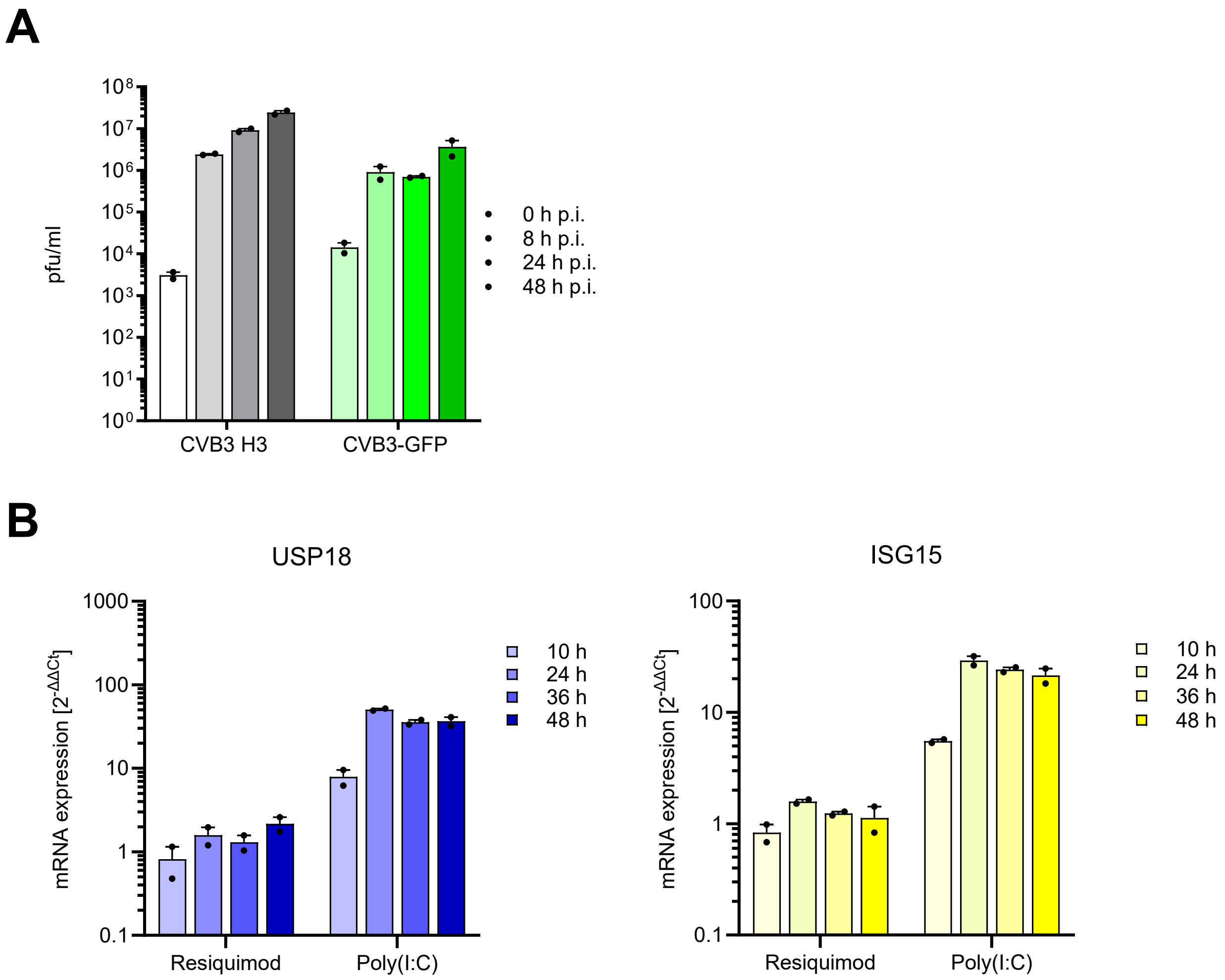

Supplemental Figure S1: Time course of virus replication and immune stimulation of primary neuronal cell cultures.

A: Neuronal cells were infected with wild-type CVB3 H3 and CVB3-GFP at MOI 0.5, and infectious particles were quantified by plaque assay at the indicated time points. **B:** Neuronal cell cultures were treated with Poly(I:C) (50 µg/ml) or Resiquimod (10 µg/ml) and mRNA expression of USP18 and ISG15 was analyzed at different time points after stimulation by quantitative RT-PCR analysis. All data were plotted as individual points, and results were presented as mean ± SEM.

Supplemental Figure S2

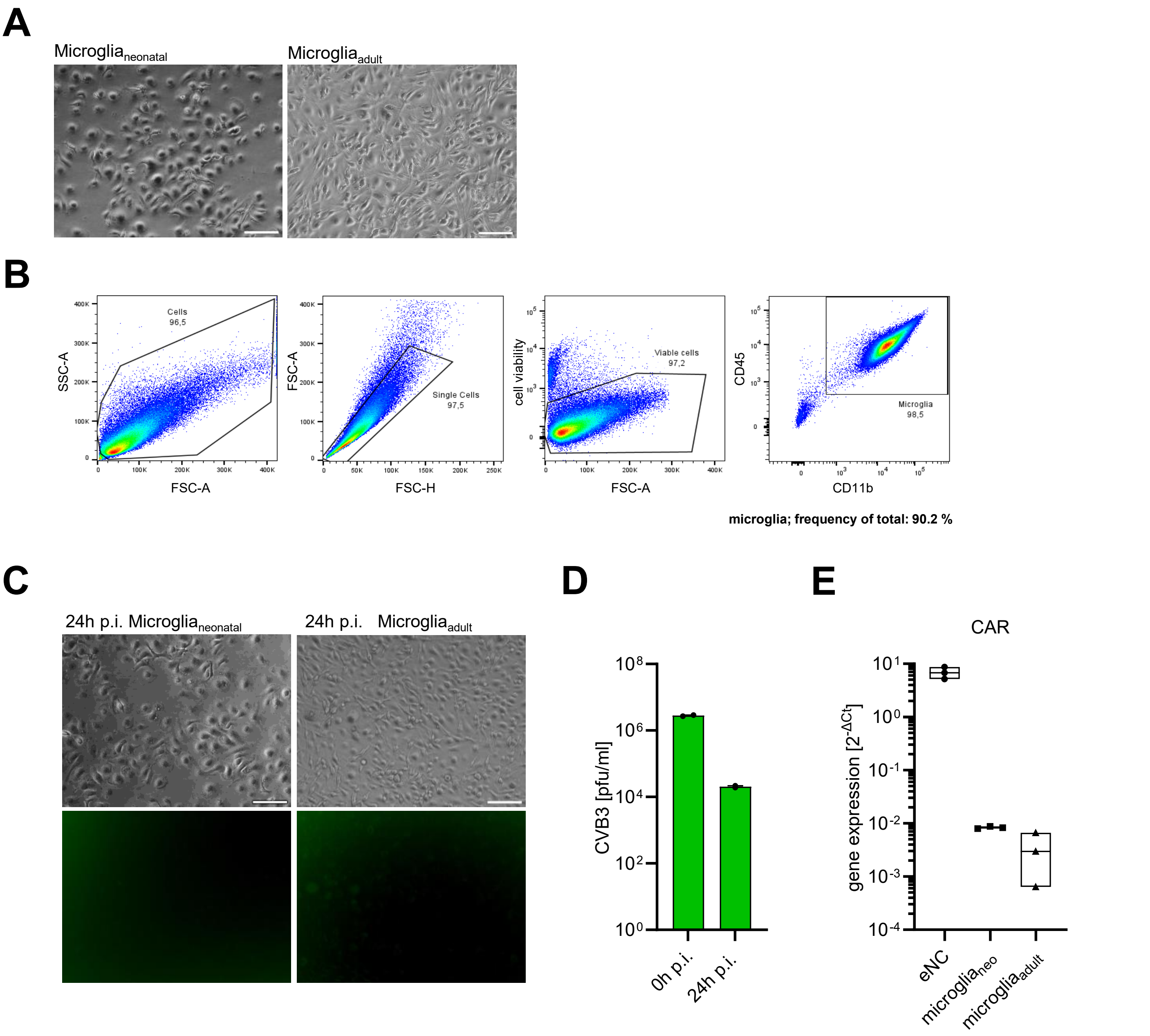

Supplemental Figure S2: Primary Neonatal and Adult Microglia Cultures: Viability, CVB3 Susceptibility and Expression Level of the CVB3 Receptor, *Coxsackievirus and Adenovirus Receptor (Car)*

A: Images of neonatal microglia (Microglia$_{neonatal}$), and adult microglia isolated from 6- to 8-week-old mice (Microglia$_{adult}$) 24 h after replating. Scale bar (100 µm) is indicated. **B:** Analysis of cell viability of adult microglia cultures by flow cytometry analysis following harvesting. **C:** Virus replication in primary microglia infected with CVB3 (MOI 0.5, 5 or 50) was assessed at 24 h post-infection by GFP expression (scale bar = 100 µm) and **D:** viral progeny quantification via plaque assay. **E:** mRNA expression of the Coxsackievirus receptor protein *Car* in primary neuronal cells (eNC) and the two microglia cultures was analyzed by quantitative RT-PCR (n=3). All data were plotted as individual points, and results were presented as mean ± SEM.

Supplemental Figure S3

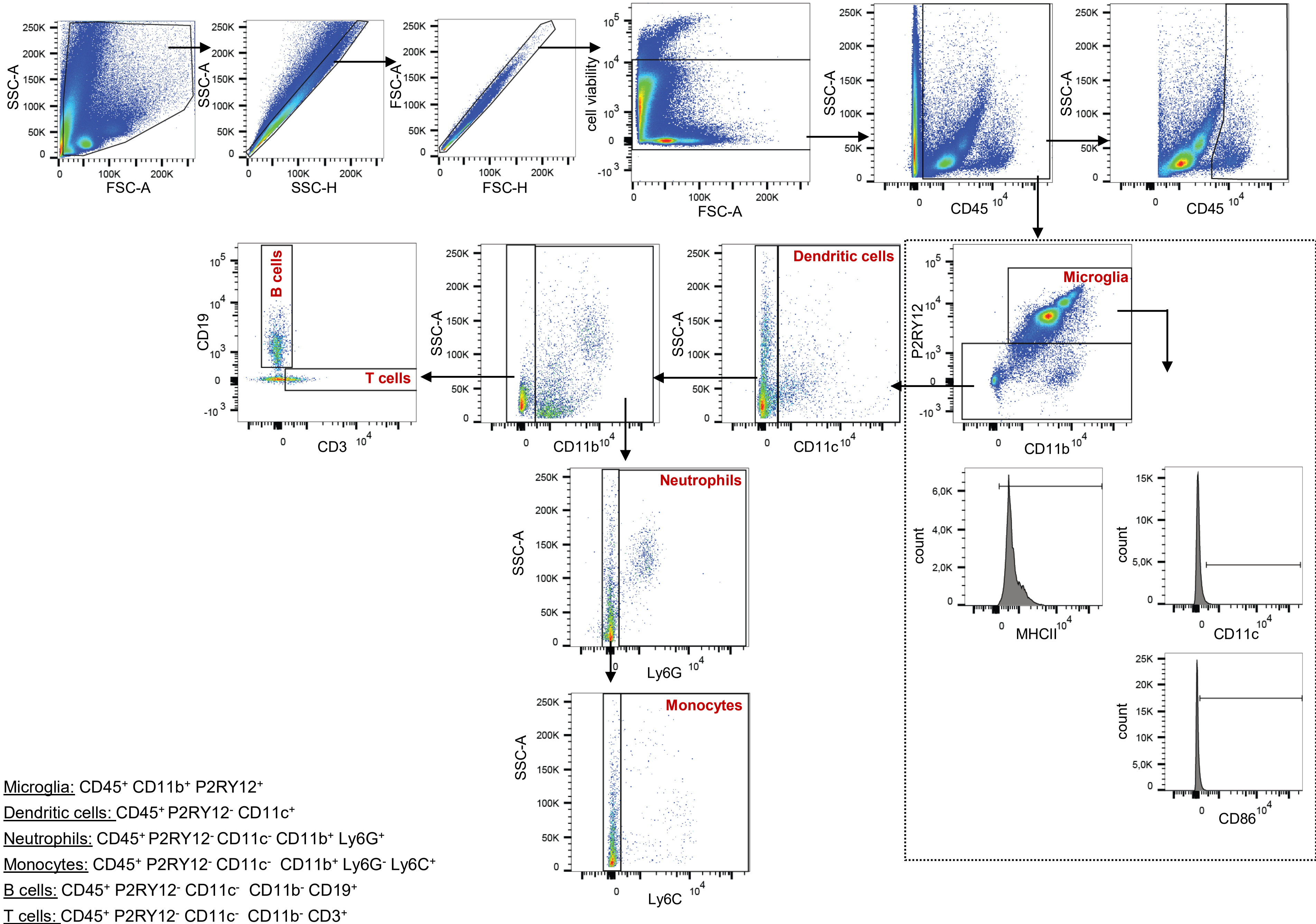

<u>Microglia:</u> CD45$^+$ CD11b$^+$ P2RY12$^+$

<u>Dendritic cells:</u> CD45$^+$ P2RY12$^-$ CD11c$^+$

<u>Neutrophils:</u> CD45$^+$ P2RY12$^-$ CD11c$^-$ CD11b$^+$ Ly6G$^+$

<u>Monocytes:</u> CD45$^+$ P2RY12$^-$ CD11c$^-$ CD11b$^+$ Ly6G$^-$ Ly6C$^+$

<u>B cells:</u> CD45$^+$ P2RY12$^-$ CD11c$^-$ CD11b$^-$ CD19$^+$

<u>T cells:</u> CD45$^+$ P2RY12$^-$ CD11c$^-$ CD11b$^-$ CD3$^+$

Supplemental Figure S3: Gating Strategy of Isolated Immune Cells from Brain Tissue.
Immune cells were isolated from brain tissue, stained with fluorochrome-linked antibodies and measured with flow cytometry. The corresponding gating strategy is depicted. Cells were differentiated from debris by an FSC/SSC gate. Subsequently, cell doublets were eliminated by SSC-A/SSC-H and FSC-A/FSC-H gating. Staining with a fixable cell viability dye separated living cells from dead cells. Immune cell subpopulations were identified by combinations of cell surface markers as indicated.

Supplemental Figure S4

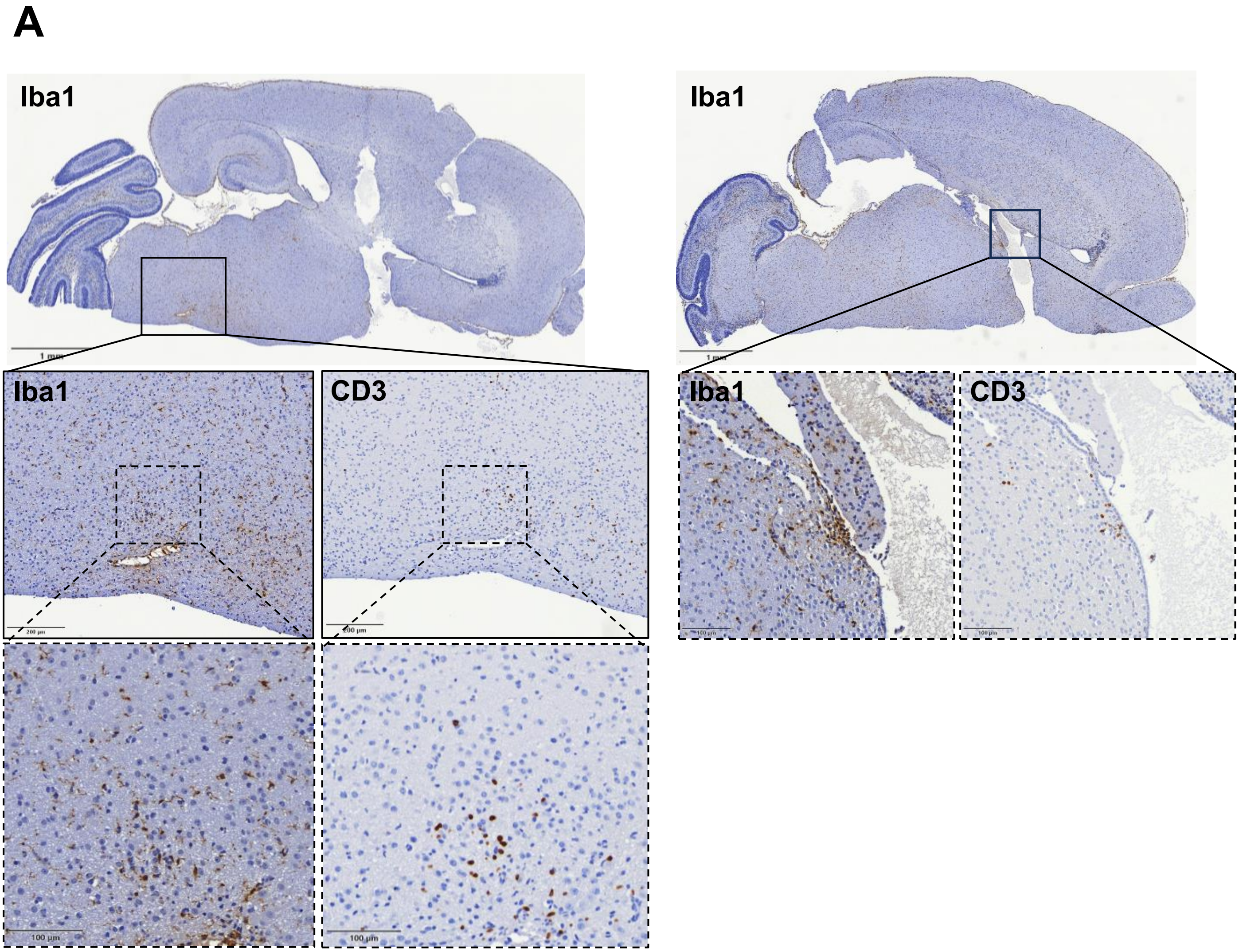

Supplemental Figure S4

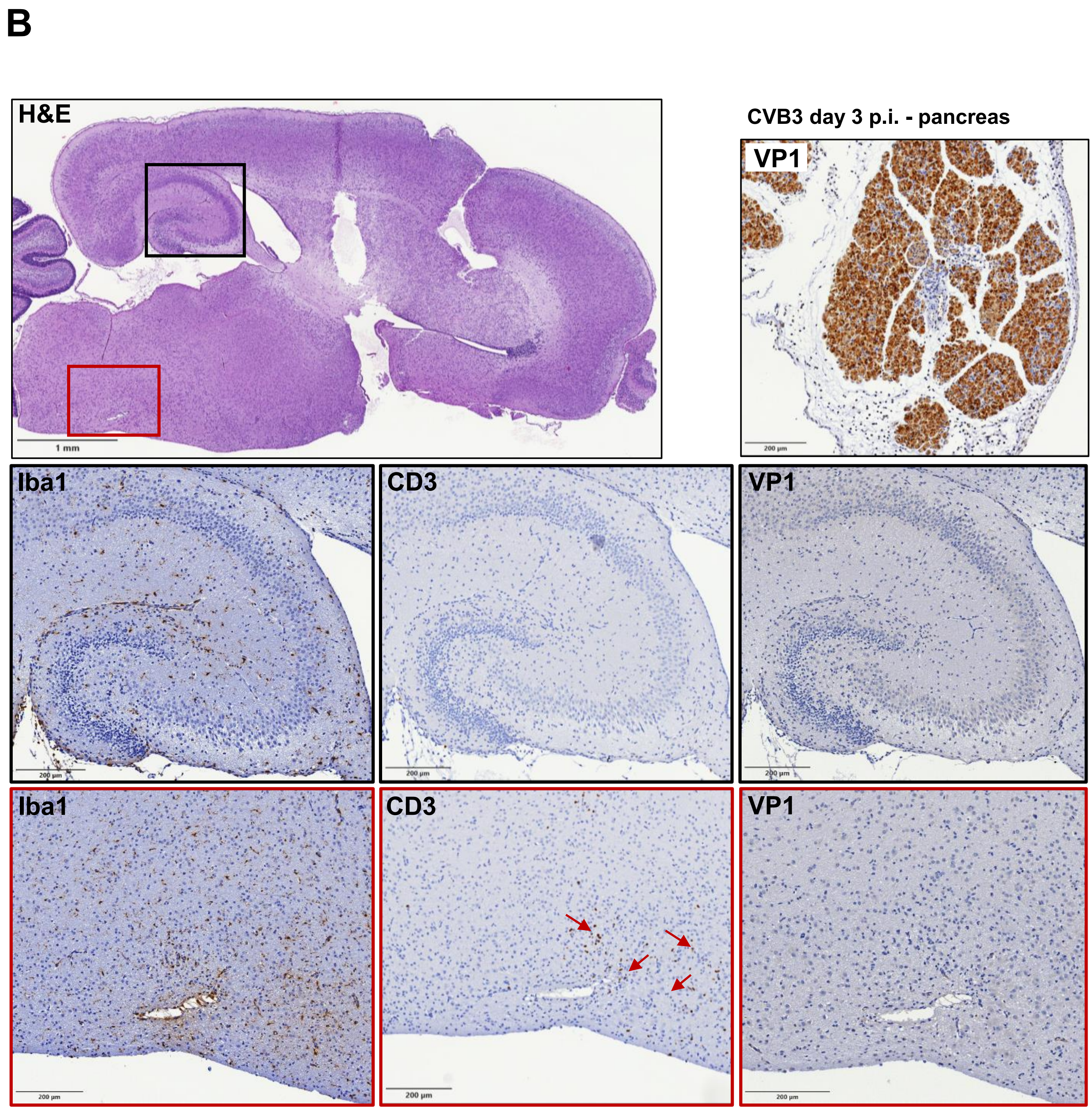

Supplemental Figure S4: Histological analysis of mice brains on day 5 after infection with CVB3-GFP. A: Immunohistochemistry was performed on brain sections using Iba1 or CD3 (dilution 1:100, cat. MCA1477, Biorad). Imaging was performed on a Zeiss Axioscan 7 system equipped with a 20x objective. Brains of two infected mice are shown. Magnified views of representative regions highlight T cell infiltrates and Iba1^{+} microglia (top row: scale bar = 1 mm, middle row: scale bar right = 100 µm, scale left = 200 µm, lower row: scale bar = 100 µm). **B:** Immunohistochemistry on brain sections using Iba1, CD3 (dilution 1:100, Biorad) and VP1 (1:500, mediagnost). Two regions showing activated microglia or the presence of CD3^{+} T cells (hippocampus in black; midbrain in red) were also examined for cells expressing the viral protein 1 (VP1); top row: scale bar = 1 mm, middle and lower row: scale bar = 100 µm. As positive control for VP1 staining, pancreas tissue of adult mice 3 days after intraperitoneal injection was used (scale bar = 200 µm).

Supplemental Figure S5

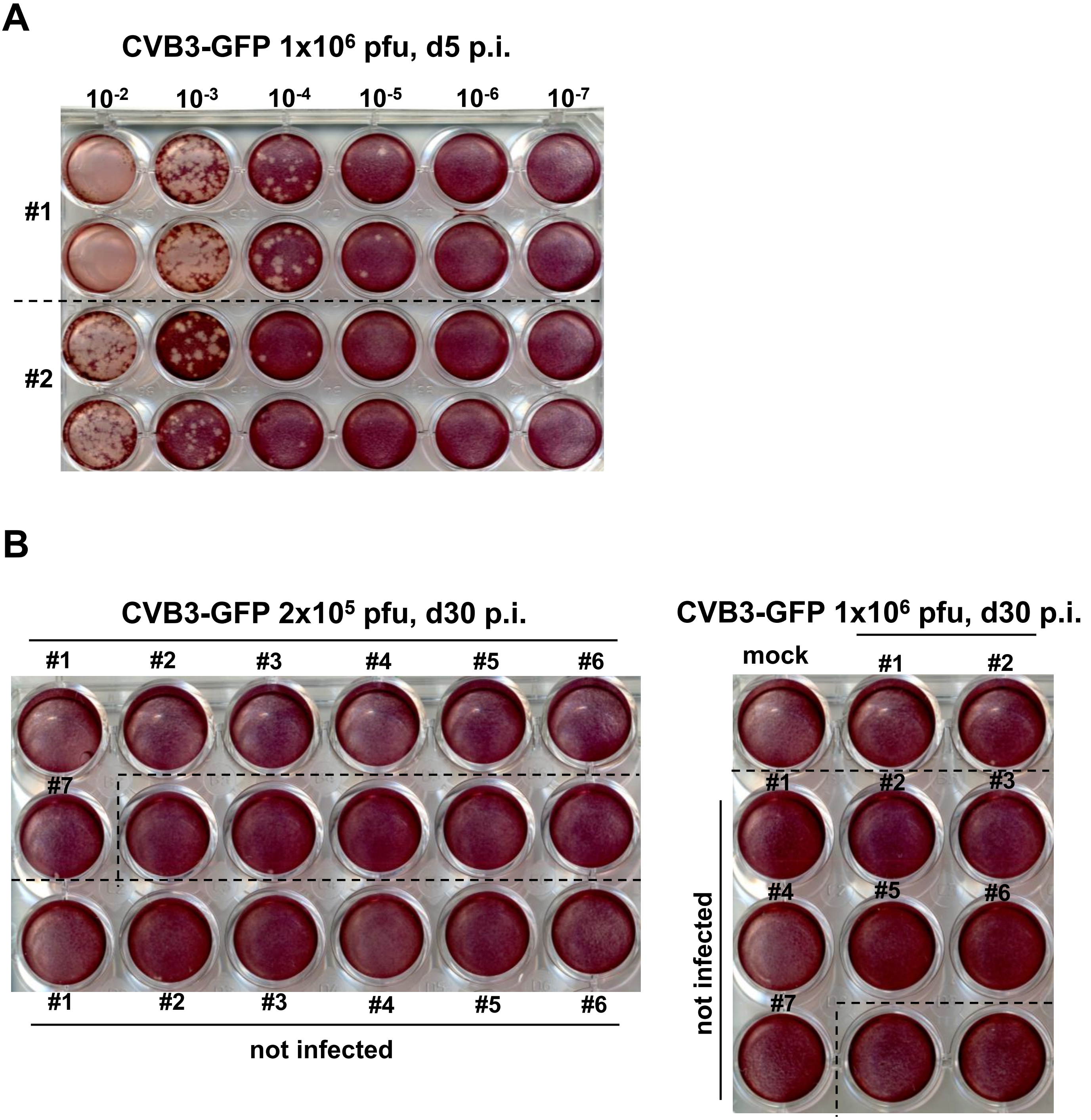

Supplemental Figure S5: Plaque-assay of brain tissues from mice on day 5 and day 30 post-infection. Brain tissue of mice on day 5 (**A**) and 30 (**B**) after intracerebroventricular CVB3 injection was homogenized in Eagle's minimal essential medium without FCS and subsequently frozen and thawed. **A:** Plaque assay of two infected mouse brain tissues (1x10^6 pfu CVB-GFP, day 5 p.i.) were shown as examples **B:** Plaque assay of brain tissue from CVB3-infected mice on day 30 p.i. revealed no infectious virus in the brain. Plaques assay was done with un-diluted brain homogenate. 1x10^6 pfu CVB-GFP, day 30 p.i.: infected mice n=2, not infected controls n=7; 2x10^5 pfu CVB-GFP, d30 p.i.: infected mice N=7, not infected (mock) controls n=6.

Supplemental Figure S6

Figure 1A

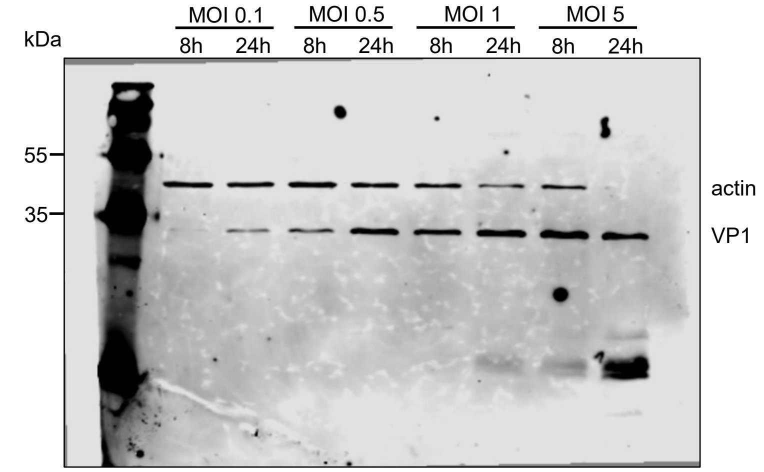

Figure 1D

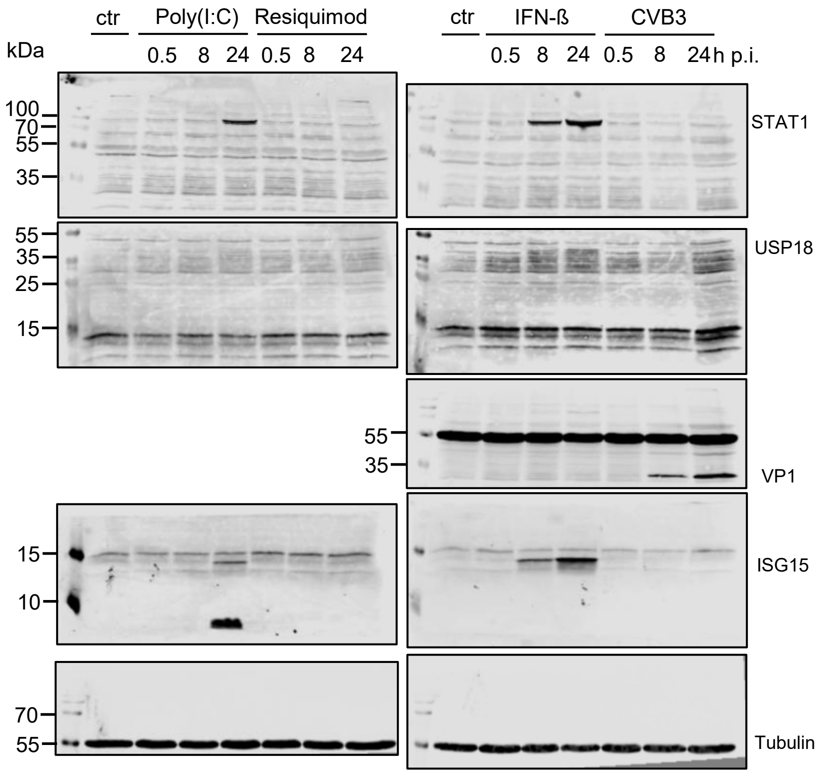

Figure 2B

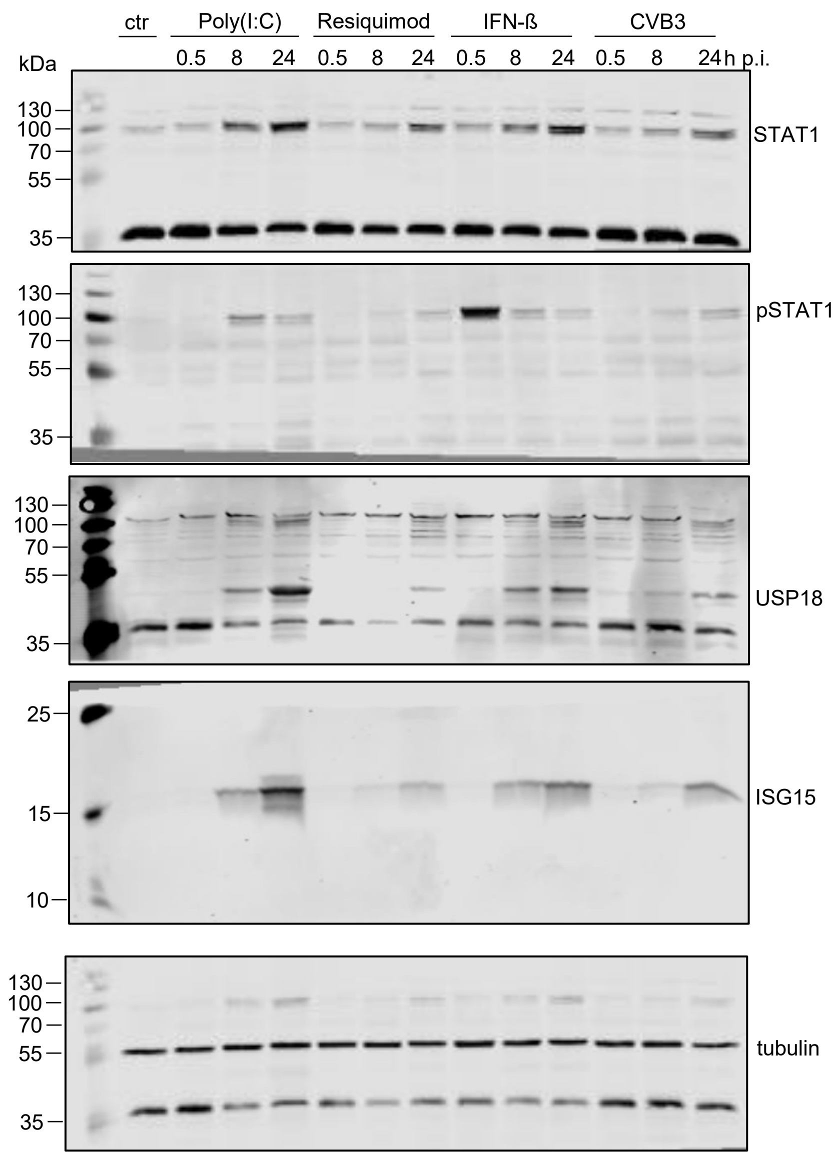

Figure 2C

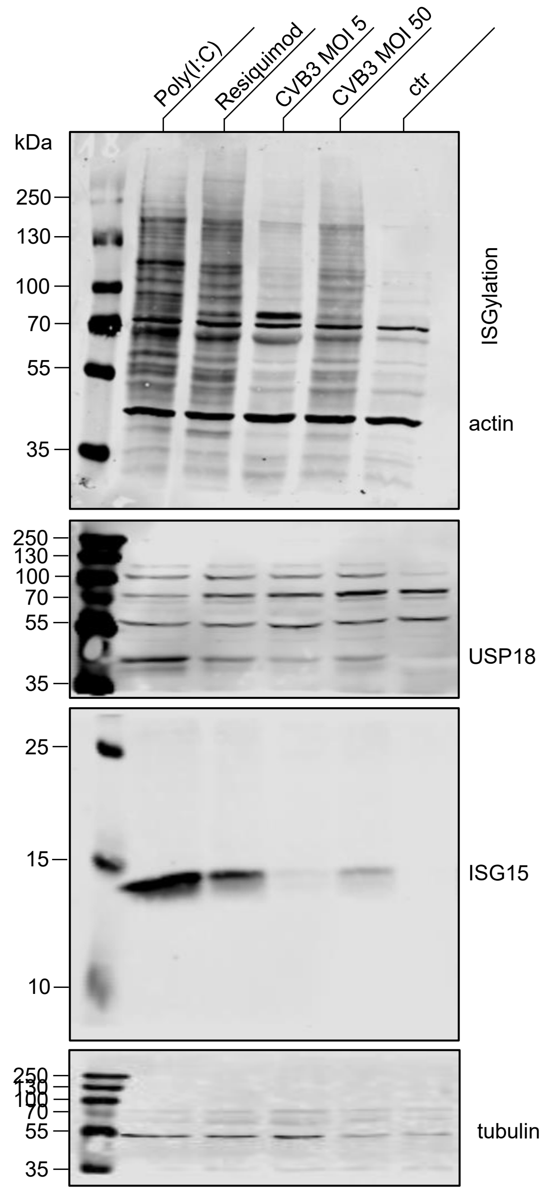

Figure 4E

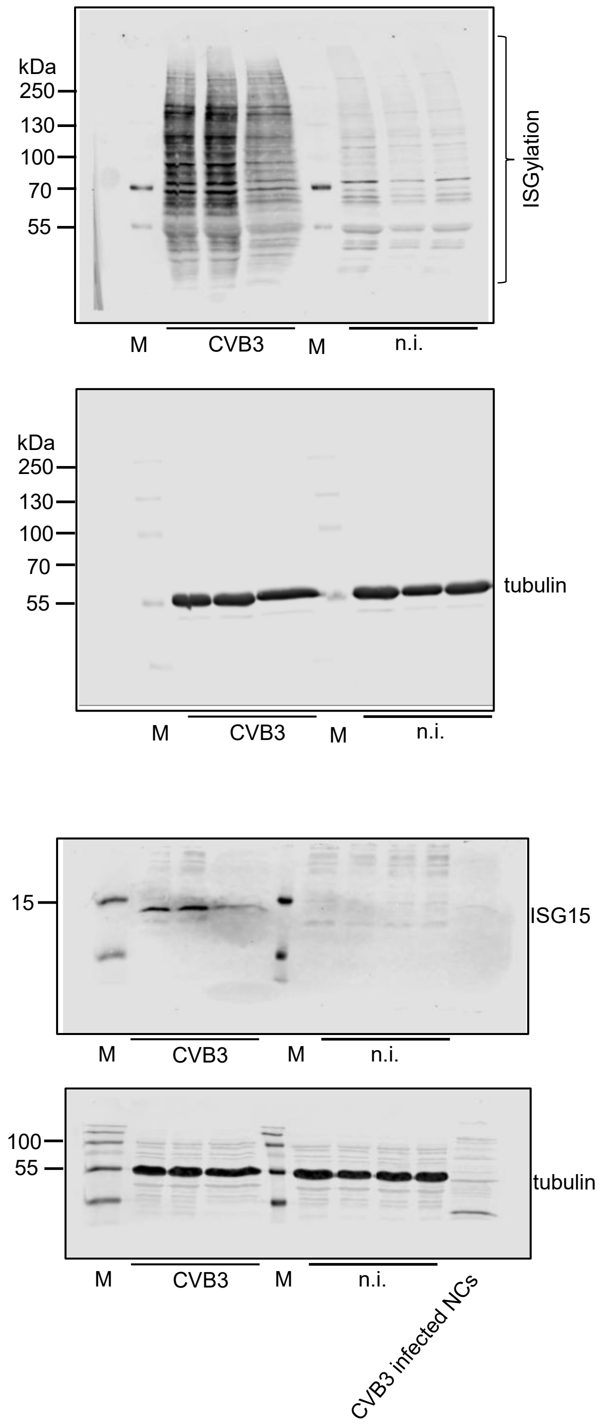

Supplemental Figure S6: Original Western Blots.
Cells were treated as described in detail in the respective figure legends. Cells and brain tissues were lysed and expression and/or phosphorylation status of various proteins, as well as ISGylation was analyzed via immunoblotting described in the Material and Methodes section. Proteins were visualized using an Odyssey CLx infrared system (LI-COR biotechnology) and the Image Studio Lite software version 5.2 (LI-COR biotechnology).
